# Single-cell sequencing reveals that endothelial cells, EndMT cells and mural cells contribute to the pathogenesis of cavernous malformations

**DOI:** 10.1038/s12276-023-00962-w

**Published:** 2023-03-13

**Authors:** Jian Ren, Xiao Xiao, Ruofei Li, Cheng Lv, Yu Zhang, Leiming Wang, Tao Hong, Hongqi Zhang, Yibo Wang

**Affiliations:** 1 Department of Neurosurgery, Xuanwu Hospital, Capital Medical University, China International Neuroscience Institute, Beijing, China; 2 State Key Laboratory of Cardiovascular Disease, Fuwai Hospital, National Center for Cardiovascular Diseases, Chinese Academy of Medical Sciences and Peking Union Medical College, Beijing, China; 3 Department of Pathology, Xuanwu Hospital, Capital Medical University, China International Neuroscience Institute, Beijing, China

**Keywords:** Computational biology and bioinformatics, Cerebrovascular disorders

## Abstract

Cavernous malformations (CMs) invading the central nervous system occur in ~0.16–0.4% of the general population, often resulting in hemorrhages and focal neurological deficits. Further understanding of disease mechanisms and therapeutic strategies requires a deeper knowledge of CMs in humans. Herein, we performed single-cell RNA sequencing (scRNA-seq) analysis on unselected viable cells from twelve human CM samples and three control samples. A total of 112,670 high-quality cells were clustered into 11 major cell types, which shared a number of common features in CMs harboring different genetic mutations. A new EC subpopulation marked with PLVAP was uniquely identified in lesions. The cellular ligand‒receptor network revealed that the PLVAP-positive EC subcluster was the strongest contributor to the ANGPT and VEGF signaling pathways in all cell types. The PI3K/AKT/mTOR pathway was strongly activated in the PLVAP-positive subcluster even in non-*PIK3CA* mutation carriers. Moreover, endothelial-to-mesenchymal transition (EndMT) cells were identified for the first time in CMs at the single-cell level, which was accompanied by strong immune activation. The transcription factor SPI1 was predicted to be a novel key driver of EndMT, which was confirmed by in vitro and in vivo studies. A specific fibroblast-like phenotype was more prevalent in lesion smooth muscle cells, hinting at the role of vessel reconstructions and repairs in CMs, and we also confirmed that TWIST1 could induce SMC phenotypic switching in vitro and in vivo. Our results provide novel insights into the pathomechanism decryption and further precise therapy of CMs.

## Central nervous system: Understanding what causes cavernous malformations

Sequencing all the RNA in single cells has provided clues to the development of cavernous malformations (CMs), small clusters of thin-walled misshapen blood vessels in the brain or nervous system. CMs can result in hemorrhage or neurological symptoms, and their causes are poorly understood. A team led by Yibo Wang at Peking Union Medical College and Hongqi Zhang and Tao Hong at Capital Medical University in Beijing, China, sequenced all the RNA in over 100,000 single cells from samples from CM patients and healthy controls, allowing them to identify molecular events that can lead to CMs. They demonstrated how the gene expression landscape is altered, especially in ECs, EndMT cells and mural cells. These results provide a strategy for investigating the genetic and molecular causes of CMs, and may help in identifying new therapies.

## Introduction

Cavernous malformations (CMs) are among the most prevalent sporadic and familial (or inherited) vascular malformations invading the central nervous system (CNS) and occur in ~0.16–0.4% of the general population, often leading to hemorrhages and focal neurological deficits^1–3^. Over 80% of CMs occur sporadically^4^. Our previous work identified activating *MAP3K3* and *PIK3CA* somatic mutations in the majority (90.1%) of sporadic CMs of the CNS^1^. Patients with familial CMs harbor biallelic loss-of-function (LOF) mutations in one of three cerebral cavernous malformation (CCM)-associated genes: Krev interaction trapped 1 (*KRIT1*, also called *CCM1*), *CCM2*, and programmed cell death 10 (*PDCD10*, also called *CCM3*), which encode a heterotrimeric CCM protein complex affecting endothelium stabilization^4,5^.

Genetic studies have supported sporadic and familial CCMs following a double-hit model^5–7^. Gain-of-function mutations in *MAP3K3* and LOF in CCM-associated genes lead to similar functional consequences in initiating CCM formation, distinguished from *PIK3CA* mutations, which aggravate lesion growth but are not indispensable for lesion formation^5^. Mutations in *PIK3CA* and CCM-associated genes lead to cavernoma development via a cancer-like mechanism. *PIK3CA* is considered to be a vascular “oncogene” capable of driving excess vascular growth, while CCM-associated genes play opposite roles in suppressing vessel growth^8^. To date, the systemic transcriptomic profile in CMs with different mutations has not been demonstrated.

The growth of vascular malformations involves multiple cell types, such as ECs and smooth muscle cells (SMCs)^1,9–11^. Cellular heterogeneity is critical for a detailed understanding of the pathogenesis of CMs. Although some causative genetic mutations of CMs have been identified in succession^1,4–6,8^, the transcriptomic heterogeneity and molecular profiles of individual cells are poorly understood. Single-cell RNA sequencing (scRNA-seq) technology now allows the characterization of gene expression in large numbers of individual cells^12^, making it possible to identify and probe the cellular subpopulations and heterogeneity in CMs. Recently, scRNA-seq has been applied to map EC diversity in the CCMs of *Pdcd10*-deletion mice^13^. However, a comprehensive cellular atlas of CMs in the CNS has yet to be reported.

In this study, we used scRNA-seq analysis and constructed an unbiased and systemic transcriptomic landscape of CNS CMs that harbored distinct genetic mutations. We defined eleven major cell types and investigated multiple molecular targets in different cell populations, which lay a basis for further clinical precision therapy for CMs.

## Materials and methods

The authors declare that all supporting data are available within the article and its online Supplemental Material.

### Study populations

CM samples obtained from 12 patients were used for single-cell analysis. All included patients underwent surgical resection at Xuanwu Hospital, Capital Medical University, China, between May 2017 and January 2021. The diagnosis of CMs was based on the typical appearance of the lesion on MR imaging and pathological examination of resected samples. The control samples were temporal lobe or frontal lobe tissues obtained from patients with epilepsy. The study was approved by the ethics committee of Xuanwu Hospital (NO.2016032), and written informed consent was obtained from all patients or their guardians before surgery.

### Single-cell data analysis

ScRNA-seq was performed by CapitalBio Technology (Beijing, China). Quality control, dimension reduction, and clustering of scRNA-seq data were performed using the Seurat R package (version 4.0.5)^14^. Low-quality cells (i.e., those expressing <200 or >7000 genes and with >25% expression of mitochondrial genes) were removed. Filtered data were normalized (normalization.method = “LogNormalize”) with a scaling factor of 10,000 and scaled by regressing out the total unique molecular identifier counts and mitochondrial gene percentages. Highly variable genes were selected with the FindVariableFeatures function and used for the next principal component analysis (PCA). The top 10 significant PCs were selected to reduce the dimension, and the Seurat FindClusters function was used for cell type clustering (resolution = 0.12). Then, uniform manifold approximation and projection (UMAP) analysis was performed for dimension reduction and visualization in two-dimensional maps^15^. Eleven cell types were defined with typical marker genes (Supplementary Table 1). Subsequently, ECs (marked by *PECAM1* and *VWF* expression) were selected and reclustered into 4 subclusters (resolution = 0.20). Marker genes of each EC subcluster were identified using the Seurat FindAllMarkers function (Wilcoxon rank sum test, min.pct = 0.25, logfc.threshold = 0.25)^16^. Mural cells (marked *ACTA2*, *MYH11*) were reclustered using the same methods as ECs.

### Public scRNA data analysis

We obtained public raw gene expression matrices from Orsenigo F et al. (GSE155788)^13^ in the Gene Expression Omnibus database, whose overall design was scRNA-seq of brain ECs from *Pdcd10*-KO and *Pdcd10*-WT mice (2 mice per group). Data analysis was based on Seurat (version 4.0.5). Low-quality cells (i.e., those expressing <2000 or >7000 genes and with >25% expression of mitochondrial genes) were filtered out. The expression matrices of the remaining cells were processed according to standard procedures: normalization, scaling, dimension reduction, and cell type clustering (resolution = 0.20)^14^. ECs (marked by *Pecam1* and *Cdh5* expression) were selected for subsequent analysis.

### Pseudotime trajectory analysis

The cellular differentiation trajectory was constructed with the Monocle 2 R package (version 2.20.0)^17^. The DifferentGeneTest function was used for differential expression analysis, and the top 2000 significant genes were selected to define cell progression. The DDRTree method was used to reduce the dimensions. The pseudotime trajectory was visualized with the plot_cell_trajectory function in the Monocle 2 package.

### Cell‒cell communication analysis

To explore the cellular interactions between different cell types, we performed cell‒cell communication analysis using the CellChat R package (version 1.4.0)^18^. The signaling pathways were visualized with the netAnalysis_signalingRole_scatter function.

### SCENIC analysis

To identify transcription factors regulated in specific cell types, we employed single-cell regulatory network inference and clustering (SCENIC) analysis for enrichment prediction^19^. Normalized expression matrices were inputted, and coexpression networks were constructed using the GENIE3 R package (version 1.16.0). RcisTarget (version 1.14.0) was then used to refine the network modules by identifying the transcription factor binding motifs. After constructing the regulons, AUCell (version 1.16.0) scored individual cells using the “area under the curve (AUC)” to calculate the enrichment rank of transcription factor target genes in the cell signatures and evaluate the regulon activities for downstream analysis and visualization.

### Enrichment analysis

Gene Ontology (GO) and Kyoto Encyclopedia of Genes and Genomes (KEGG) enrichment analyses were performed using the clusterProfiler R package (version 4.0.5)^20^. Gene set enrichment analysis (GSEA) was based on the Molecular Signatures Database (http://www.gsea-msigdb.org/gsea/index.jsp) or self-defined gene sets composed of representative genes enriched in GO and KEGG analyses^21^. Gene set scores were computed with the Seurat AddModuleScore function, also based on self-defined gene sets^14^.

### Mouse treatment

All animal use and welfare adhered to the National Institutes of Health’s Guide for the Care and Use of Laboratory Animals following a protocol reviewed and approved by the State Key Laboratory of Cardiovascular Disease, National Center for Cardiovascular Diseases (Beijing, China; permit number: 0000869). All animals were housed in standard cages in a temperature- and humidity-controlled environment on a 12 h/12 h light/dark cycle with free access to water.

Two-month-old C57BL/6 J mice were obtained from the Nanjing Biomedical Research Institute of Nanjing University and randomly assigned into experimental and control groups by drawing lots. Adeno-associated virus (AAV) with the TIE promoter driving SPI1 packaged in the AAV9 capsid (AAV9-TIE-SPI1) (GeneChem Co., Shanghai) and AAV with the SM22a promoter driving TWIST1 packaged in the AAV9 capsid (AAV9-SM22a-TWIST1) (GeneChem Co., Shanghai) were injected into the brains of 2-month-old C57BL/6 J mice. Briefly, mice were anesthetized and stereotactically injected with 2 μl of AAV (2 × 10^9^ viral genome (vg)) into the right basal ganglia 3 mm below the cortex. Control mice received 2 μl of AAV9-TIE-vector or AAV9-SM22a-vector (2 × 10^9^ vg). Mice were sacrificed for subsequent analysis after 7 days.

### Primary cell isolation

Brain primary endothelial and SMCs of mice were selected using a CD31 MicroBeads mouse kit (130-097-418, Miltenyi) and a CD146 MicroBeads mouse kit (130-092-007, Miltenyi) according to the manufacturer’s protocols. Freshly resected brain tissues were dissected mechanically and isolated using type II collagenase to prepare single-cell suspensions. Subsequently, 10 µL of MicroBeads per 1 × 10^7^ total cells was added for magnetic labeling, and then magnetic separation was performed. CD146 MicroBeads could collect ECs and SMCs, so CD31 MicroBeads selection was first performed, and then CD31-negative cell suspensions were sorted with CD146 MicroBeads to obtain SMCs. Isolated primary cells were immediately used for subsequent analysis.

### Cell culture and plasmid transfection

HUVECs and HASMCs were cultured in ECM (endothelial cell medium) (Sciencell, San Diego, CA) and SMCM (smooth muscle cell medium) (Sciencell) after processing with 10% fetal bovine serum, 100 U/mL penicillin, and 100 mg/mL streptomycin in each medium at 37 °C in 5% CO_2_.

Plasmids were purchased from Shanghai GeneChem Co., Ltd. The open reading frames were amplified using primers that included an added XhoI site on the 5′ end and a KpnI site on the 3′ end. PCR amplicons were then subcloned into CV702 using the XhoI/KpnI sites.

Cells were used between passages 5 and 7, grown in 6-well cell culture plates (Costa) and transfected with 2 µg plasmid DNA per well using Lipofectamine TM 3000 (Invitrogen, #L3000008) according to the manufacturer’s protocol.

### Immunohistochemical staining

Fresh tissues were fixed, embedded in paraffin, and sectioned into 5 μm thick slices. After deparaffinization, sections were heated in citrate buffer (0.01 M, pH = 6) for 30 min for antigen retrieval and incubated in 3% hydrogen peroxide for 10 min to quench endogenous peroxidase activity. Subsequently, sections were incubated with primary antibodies (P-AKT (Ser473) (CST, 4060 S, 1:100); P-S6 (Ser235/236) (CST, 4858 S, 1:500); P-S6 (Ser240/244) (CST, 5364 S, 1:800)) and suitably diluted secondary antibodies and visualized using diaminobenzidine chromogenic solution (Abcam). Immunohistochemistry scores were determined by multiplying the pathologist-assessed IHC intensity by the fraction of positive cells^4^.

### Immunofluorescence staining

Immunofluorescence staining was conducted to verify the coexpression of target proteins in CM lesions. Sections were blocked and permeabilized with 2% goat serum (Sigma) and 0.1% Triton X-100 (Sigma) in PBS buffer for 1 h and incubated in primary antibodies (CD31 (CST, 77699 S, 1:200); PLVAP (Abcam, ab81719, 1:200); P-AKT (Ser473) (CST, 4060 S, 1:500); P-S6 (Ser235/236) (CST, 4858 S, 1:100); a-SMA (CST, 19245, 1:200); Lumican (Abcam, ab168348, 1:200)) overnight at 4 °C. Subsequently, sections were successively washed three times with PBS and incubated in secondary antibodies for 1 h. Nuclei were stained using DAPI (0.5 μg/ml), and images were taken with a confocal microscope (Leica Microsystems). Positive areas were quantified using NIH ImageJ software (version 1.8.0)^1^.

### Real-time quantitative PCR

Total RNA was isolated using TRIzol reagent (Invitrogen, CA, USA). One microgram of total RNA was converted to cDNA. Real-time quantitative PCR (RT‒qPCR) primers are shown in Supplementary Table 2 and Supplementary Table 3. Each sample was run in triplicate to ensure quantitative accuracy, and the threshold cycle numbers (Ct) were averaged. The results were calculated using the 2^−ΔΔCt^ method.

### Phalloidin staining

Fluorescent phalloidin (Invitrogen, A22287) was used to observe the cellular morphology of HUVECs and HASMCs after plasmid transfection. The cells were fixed in 3.7% methanol-free formaldehyde solution, permeabilized in 0.1% Triton™ X-100 in PBS for 15 min, and then incubated in fluorescent phalloidin staining solution for 40 min at room temperature. Nuclei were stained using DAPI (0.5 μg/ml), and images were taken with a confocal microscope (Leica Microsystems).

### Statistical analysis

Marker genes (or DEGs between two groups) were identified using the Seurat FindAllMarkers (or FindMarkers) function, and *P* values were calculated with the Wilcoxon rank sum test. For pathway enrichment analysis, *P* values were computed with the hypergeometric test and adjusted in a Benjamini‒Hochberg procedure for multiple hypothesis correction. The data from the pathology verification and RT‒qPCR assays are presented as the means ± standard deviations (SDs) and were analyzed by Student’s *t* test or one-way ANOVA (>2 groups) for *P* value determination. Significance is indicated by *P < 0.05, **P < 0.01, ***P < 0.001, ****P < 0.0001. All statistical analyses were performed with R (https://www.r-project.org/), python (https://www.python.org/) or PRISM (GraphPad Software Inc).

## Results

### Global cellular landscape in the four mutation groups

**Fig. 1:**
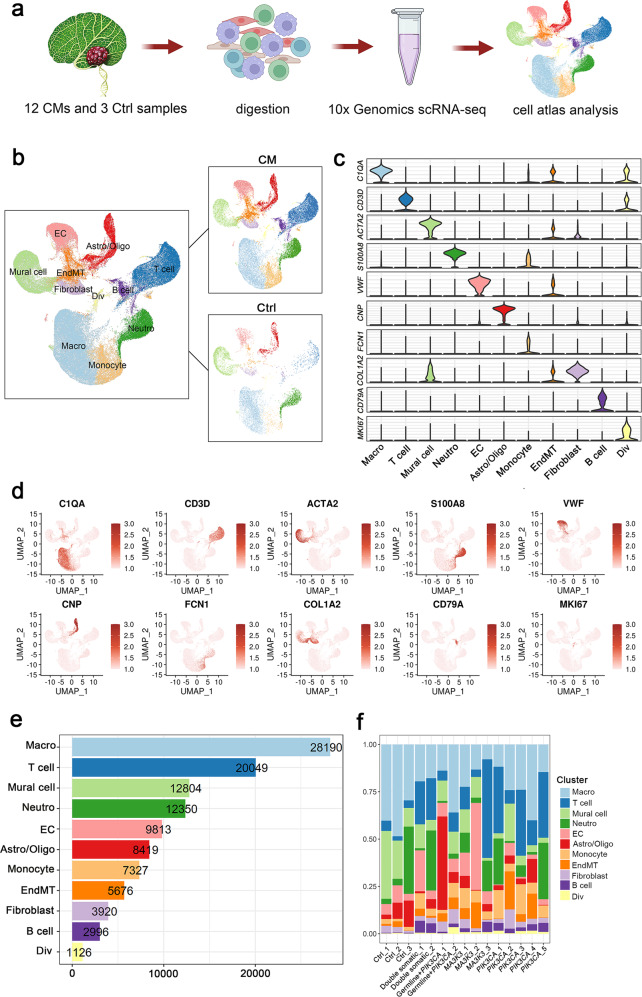
Cellular atlas of CM and control samples. **a** Schematic diagram of the scRNA-seq analysis workflow. Twelve CM and three control tissue samples were digested into single cells and sequenced using the 10x Genomics platform. **b** Uniform manifold approximation and projection (UMAP) plot for eleven cell types of 112670 high-quality single cells in control and CM samples. Marker genes for 11 distinct cell types shown in (**c**) violin plots and (**d**) UMAP feature plots. **e** Cell numbers of eleven cell types. **f** The proportion of all eleven cell types in samples. Macro macrophage, Neutro neutrophil, EC endothelial cell, Astro/Oligo astrocyte/oligodendrocyte, EndMT endothelial-to-mesenchymal transition, Div dividing immune cell.

We previously identified activating *MAP3K3* and *PIK3CA* somatic mutations in the majority (90.1%) of sporadic CMs of the CNS. To investigate the cellular diversity and molecular signatures in CM lesions, we generated scRNA-seq profiles from fifteen CNS samples (twelve CM samples and three control samples) using 10× Genomics sequencing. (Fig. 1a). The clinical characteristics of these participants were recorded at the time of recruitment (Supplementary Fig. 1). Gene mutations were screened by whole exome sequencing (WES) through next-generation sequencing, and the result was verified with ddPCR (Supplementary Fig. 1). Since the CMs of a small number of patients with sporadic CMs also carried germline mutations, we divided the genotypes of CMs into 2 categories: *PIK3CA* mutation carriers and non-*PIK3CA* mutation carriers. *PIK3CA* mutation carriers could be further divided into the *PIK3CA* mutation only group, double somatic mutation (*MAP3K3* or *CCM1* plus *PIK3CA* mutations) group, and germline mutation plus *PIK3CA* mutation group; non-*PIK3CA* mutation carriers carried only *MAP3K3* mutations. The twelve CM samples consisted of three CMs with *MAP3K3* mutations (two from the spinal cord, one from the brain), five cerebral CMs with *PIK3CA* mutations, two cerebral CMs with double somatic mutations and two cerebral CMs with germline mutations plus *PIK3CA* mutations (Table 1). It is important to note that the current study focused only on CNS CM lesions, and the sample size was larger than that of a previous study^1^.

**Table 1:** Clinical characteristics and genotype of CMs.

Part	Group-1	Group-2	Sample ID	Mutation type	Mutant-allele frequency (WES)	Age (years)	Sex	Location	Size of CMs (mm)
Brain/spinal cord	Ctrl	Ctrl	Ctrl_1	/	/	9	Male	Temporal	
			Ctrl_2	/	/	5	Female	Frontal	
			Ctrl_3	/	/	30	Female	Temporal	
	*MAP3K3* mutations	non-*PIK3CA* mutations	*MAP3K3*_1	MAP3K3 I441M	3.4%	48	Female	spinal cord (C7)	8.7
			*MAP3K3*_2	MAP3K3 I441M	8.4%	42	Female	spinal cord (T6)	12
			*MAP3K3*_3	MAP3K3 I441M	7.5%	44	Female	Pons	13.9
	*PIK3CA* mutations	with-*PIK3CA* mutations	*PIK3CA*_1	PIK3CA C420R	3.60%	29	Male	Pons	18.9
			*PIK3CA*_2	PIK3CA H1047R	5.10%	5	Female	Frontal	33
			*PIK3CA*_3	PIK3CA H1047R	1.30%	33	Male	Thalamus	23.1
			*PIK3CA*_4	PIK3CA H1047R	0.60%	31	Male	Cerebellum	17
			*PIK3CA*_5	PIK3CA H1047R	2.30%	55	Male	Pons	13.3
	Double somatic mutations		Double somatic_1	CCM1 L558Wfs*4	23.80%	37	Male	Frontal	36.8
				PIK3CA E545K	8.80%				
			Double somatic_2	MAP3K3 I441M	1.20%	30	Female	Temporal	15.5
				PIK3CA E545K	1.70%				
	Germline mutations plus *PIK3CA* mutations		Germline+*PIK3CA*_1	CCM1 K654Sfs*21	66.3%	15	Male	Frontal	26
				PIK3CA E542K	0.4%				
			Germline+*PIK3CA*_2	CCM1 L551Afs*17	55.4%	0 (10 months)	Male	Temporal	31
				PIK3CA E542K	2.3%				

Following gene expression normalization, we conducted dimensionality reduction and clustering using PCA and UMAP, respectively (Fig. 1b and Supplementary Fig. 2a, b). When analyzing the data, canonical correlation analysis in the Seurat package was used to remove batch effects during integration (Supplementary Fig. 3). With marker-based annotations^22^, 11 major cell types were identified, including macrophages (Macro), T cells, mural cells, neutrophils (Neutro), monocytes, ECs, astrocytes/oligodendrocytes (Astro/Oligo), endothelial-to-mesenchymal transition (EndMT) cells, fibroblasts, B cells and dividing immune cells (Div) (Supplementary Table 1 and Fig. 1c–e).

After stringent quality control, 112,670 cells were retained for biological analysis, which detected a median of 1707 genes and 4869 transcripts per cell (Supplementary Fig. 4). Increased percentages of monocytes and lymphocytes (T cells and B cells) were observed in CM lesions with all genotypes, suggesting an enhanced immune response in all CMs (Fig. 1f).

### Transcriptomic heterogeneity of ECs in human CM and control samples

**Fig. 2:**
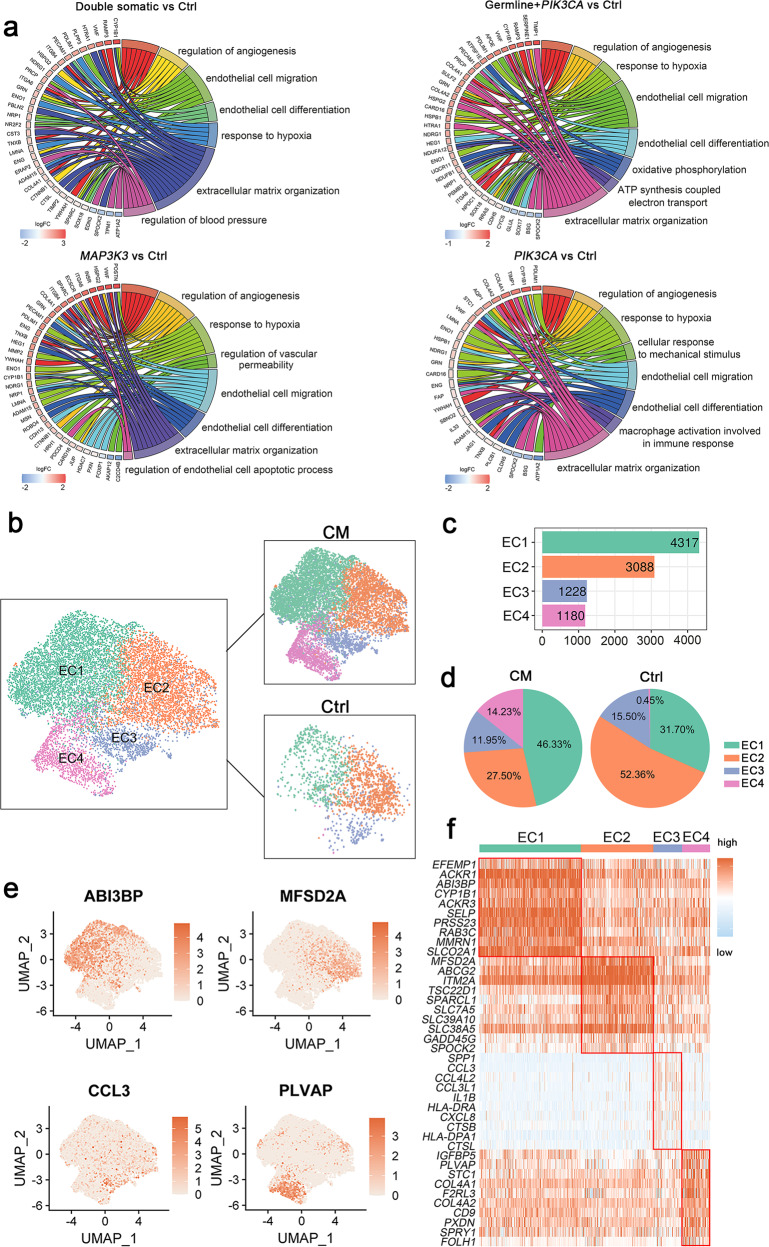
Endothelial cell clusters. **a** GO and Kyoto Encyclopedia of Genes and Genomes (KEGG) pathway enrichment analyses of differentially expressed genes (DEGs) in ECs between CM samples with different genetic mutations and control samples, shown in chord plots. **b** UMAP plot for the distributions of four EC subclusters in control and CM samples. CM, twelve CM samples; Ctrl, three control samples. **c** Cell numbers of four EC subclusters. **d** Pie plots for the cell number percentages of four EC subclusters in the control and CM groups. **e** Marker genes of four EC subclusters shown with UMAP plots. **f** Top ten differentially expressed genes (DEGs) in four EC subclusters.

A total of 9813 endothelial cells were detected in our samples. Using the identified DEGs in ECs between each mutation group and control group, we determined molecular pathways by performing gene set enrichment analysis. Gene enrichment analysis identified both commonly shared and unique molecular and cellular pathways among different mutation groups (Fig. 2a). Examples of unique pathways included regulation of blood pressure in the double somatic mutation group, ATP synthesis coupled electron transport in the germline mutation plus *PIK3CA* mutation group, the regulation of endothelial cell apoptosis in the *MAP3K3* mutation group, and macrophage activation involved in the immune response in the *PIK3CA* mutation group. Major developmental pathways known to play a critical role in endothelial homeostasis and CM progression, such as the PI3K-AKT signaling^8^, regulation of angiogenesis^23^, response to hypoxia^24^, endothelial cell migration^25^ and extracellular matrix organization^26^ pathways, were commonly found in all mutation groups.

Kahn et al. recently demonstrated that CCM-associated gene loss of function and *PIK3CA* gain of function both activated PI3K/AKT/mTOR signaling and that inhibitors targeting this signaling pathway could prevent CCM formation in mice^8^. Our results showed that single *MAP3K3* mutations (non-*PIK3CA* mutation group) can also activate PI3K/AKT/mTOR signaling (Fig. 2a and Supplementary Fig. 5), which shed light on the treatment of CMs among different mutation categories. The regulation of angiogenesis signaling^23^ and response to hypoxia^24^, which play critical roles in the development and progression of CMs, were significantly enriched in all mutation groups (Supplementary Fig. 5).

To explore EC heterogeneity and whether there would be unique EC subclusters in these CM groups, subclustering analysis of all ECs was performed. Reclustering these endothelial cells revealed 4 subclusters (Fig. 2b, c and Supplementary Fig. 6a–d). We next attempted to identify marker genes for each subcluster and annotate them.

The EC1 subcluster displayed high expression levels of extracellular matrix proteins such as ABI3BP, which plays a role in cell-substrate adhesion^27^, and EFEMP1, a member of the fibulin family widely expressed in the basement membranes of ECs^28,29^. The EC2 subcluster expressed a high level of *MFSD2A*, which regulates BBB function by regulating vesicular transcytosis across the cerebral endothelium^30^. In the absence of Mfsd2a, there was an increase in vesicular transport across the endothelial cytoplasm without an opening of the tight junction protein, leading to increased BBB leakiness^31^. In the control group, the highest proportion of Mfsd2a expression was observed in the EC2 subcluster (Fig. 2d), also reflecting that subcluster 2 had normal EC functions in maintaining the integrity of the BBB. Subcluster 3 was characterized by the expression of the immune-associated genes *CCL3*, *CCL3L1*, *CCL4L2*, *IL1B* and *CXCL8*, indicating immune response characteristics (Fig. 2e, f). Subcluster 4 exhibited a high expression level of *PLVAP* (Fig. 2e, f). PLVAP, which is involved in endothelial vesicle trafficking, is highly expressed in permeable peripheral vessels and is upregulated in CNS endothelial cells during pathological breakdown of the BBB^32^. Strikingly, the control group had almost no contribution to subcluster 4, indicating that the PLVAP-positive EC subcluster was unique in CM lesions and matched its feature of blood‒brain barrier (BBB) impairment (Fig. 2d).

### The PLVAP-positive EC subcluster was unique in CM lesions

**Fig. 3:**
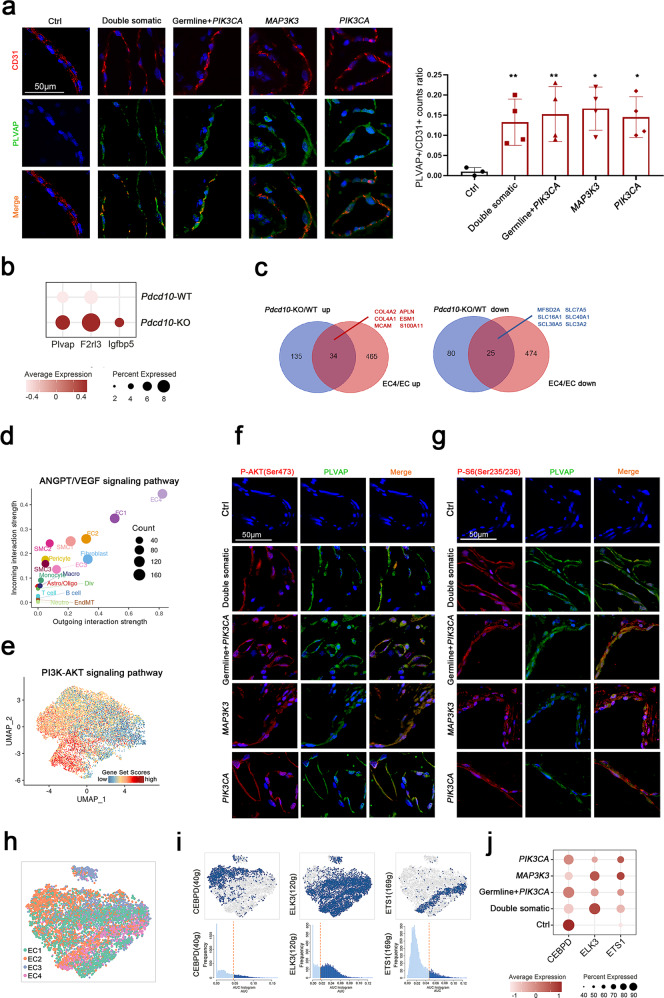
PLVAP-positive EC subcluster. **a** Immunofluorescence staining indicating the coexpression of CD31 (red) and PLVAP (green) in different groups. Scale bars, 50 µm. PLVAP + /CD31 + count ratio quantified with NIH ImageJ software (mean ± SD). There were no significant differences among the four mutation groups (one-way ANOVA), comparisons between each mutation group and the control group (Student’s *t* test), *P < 0.05, **P < 0.01. **b** Dot plot for EC4 subcluster marker genes (*Plvap, F2rl3, Igfbp5*) derived from the reanalysis of the publicly available single-cell dataset (GSE155788)^13^ comparing *Pdcd10*-KO and *Pdcd10*-WT brain ECs. **c** Venn diagrams representing the intersection between the significant differentially upregulated (left panel) or downregulated (right panel) genes in *Pdcd10*-KO vs. *Pdcd10*-WT brain ECs (blue circles) and the EC4 subcluster vs. all ECs (pink circles). The upregulated genes are written in red, and the downregulated genes are written in blue. **d** Scatter plot showing outgoing (*x*-axis) and incoming (*y*-axis) cellular interaction strength of ANGPT and VEGF signaling pathways in all cell types. **e** The distributions of the PI3K-AKT signaling pathway in four EC subclusters. Immunofluorescence staining indicating the coexpression of (**f**) P-AKT (Ser473) (red) or (**g**) P-S6 (Ser235/236) (red) with PLVAP (green) in different groups. DAPI-stained nuclei are shown in blue. Scale bars, 50 µm. **h** SCENIC-based tSNE plot for the distributions of four EC subclusters. **i** SCENIC analysis predicted transcription factors such as CEBPD, ELK3, and ETS1 as specific hubs governing the states of corresponding EC subclusters (top). Transcription factor regulon activities were quantified using AUCell (bottom). **j** Expression of CEBPD, ELK3, and ETS1 in different groups in all ECs, shown in a dot plot.

We further confirmed the presence of the PLVAP-positive EC subcluster in CM samples using immunofluorescence staining (Fig. 3a). *PDCD10* is known as one pathogenic gene of CMs, and individuals with *PDCD10* LOF mutations can develop CCMs and suffer disabling brain hemorrhages and strokes^33^. Orsenigo F et al. mapped EC diversity in *Pdcd10*-KO and *Pdcd10*-WT mice at single-cell resolution^13^. Consistent with our results, the expression of EC4 marker genes, including *Plvap*, *F2rl3* and *Igfbp5*, was also drastically upregulated in the ECs of *Pdcd10*-KO mice (Fig. 3b)^13^. We observed that the upregulated DEGs of EC4 vs. all EC subclusters and *Pdcd10*-KO vs. *Pdcd10*-WT ECs shared 34 genes (accounting for 6.8% and 20.1% of their DEGs, respectively), including *COL4A1* and *COL4A2*, which are involved in extracellular matrix organization^34^; APLN and ESM1, which participate in angiogenesis^35,36^; and MCAM and S100A11, which are critical for the inflammatory response (Fig. 3c)^37,38^. Correspondingly, downregulated DEGs shared 25 genes (accounting for 5.0% and 23.8% of their DEGs, respectively), such as *MFSD2A, SLC7A5, SLC16A1, SLC40A1, SLC38A5*, and *SLC3A2*, which are involved in BBB formation or transportation (Fig. 3c)^39,40^.

The cellular ligand‒receptor network revealed EC4 as the strongest contributor to the ANGPT and VEGF signaling pathways in all cell types, which confirmed the critical role of EC4 in angiogenesis (Fig. 3d). Functional pathway scores showed that the strongest activation of PI3K-AKT pathway signaling occurred in subcluster 4, followed by subclusters 1 and 3, with little activation in subcluster 2, in line with the features of normal ECs (Fig. 3e). The PI3K/AKT/mTOR pathway was strongly activated in the PLVAP-positive subcluster even in non-*PIK3CA* mutation carriers, which was also validated by immunofluorescence staining (Fig. 3f, g).

In addition, we applied the SCENIC algorithm to assess which transcription factors underlie differences in expression among the four subclusters of ECs (Fig. 3h). SCENIC analysis revealed that CEBPD was the predominant transcription factor in the EC2 subcluster; CEBPD has been reported to participate in the neovascularization of ECs^41^. The activity of the transcription factor ELK3 was enriched in the EC1, EC3 and EC4 subclusters. Interestingly, the ETS1 regulon was specific to the EC4 subcluster (Fig. 3i, j and Supplementary Fig. 7).

### An endothelial-to-mesenchymal transition cell type was identified, which was accompanied by immune activation

**Fig. 4:**
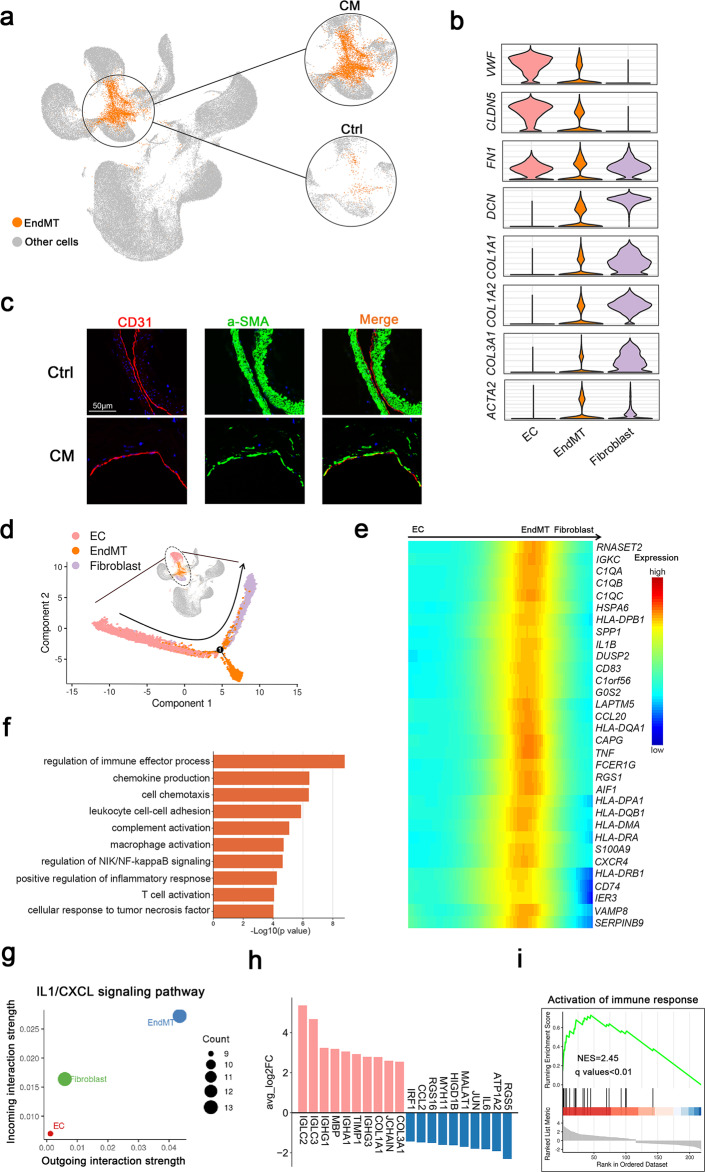
Endothelial-to-mesenchymal transition cell cluster. **a** UMAP plot for EndMT and other cells (left panel) divided into control and CM groups (right panel). **b** Marker genes of ECs, EndMT cells and fibroblasts shown in violin plots. **c** Immunofluorescence staining of EndMT markers (CD31 and a-SMA) in the control and CM groups. CD31, red; a-SMA, green and DAPI-stained nuclei shown in blue. Scale bars, 50 µm. **d** Monocle 2 pseudotime analysis for ECs, EndMT cells and fibroblasts. **e** Heatmap showing the expression of dynamic immune genes along the pseudotime axis and a bar plot for Gene Ontology (GO) enrichment analysis of these genes (**f**). **g** Scatter plot showing outgoing (*x*-axis) and incoming (*y*-axis) cellular interaction strength of IL1 and CXCL signaling pathways in ECs, EndMT cells, and fibroblasts. **h** Top ten differentially expressed genes (DEGs) upregulated (pink, avg_log2FC ≥ 0.25 and *P* value < 0.05) or downregulated (blue, avg_log2FC ≤ -0.25 and *P* value < 0.05) in lesion EndMT cells. **i** Gene set enrichment analysis (GSEA) indicating the enrichment of immune activation in lesion EndMT cells based on the Molecular Signatures Database (http://www.gsea-msigdb.org/gsea/index.jsp).

EndMT is a process characterized by the downregulation of endothelial-specific markers such as CD31 and the upregulation of mesenchymal markers such as a-SMA, and EndMT occurs in a broad spectrum of conditions, including tissue fibrosis, cancer, heterotopic ossification and atherosclerosis^42,43^. Recent evidence suggests that EndMT plays a critical role in the pathophysiology of CCMs^44^. Endothelial-specific disruption of the *Ccm1* gene in mice induces EndMT, which activates the TGF-β and bone morphogenetic protein signaling pathways and contributes to the development of vascular malformations^45^. However, the findings of previous studies of EndMT may be confounded by other cells in whole tissues, and the detailed characteristics and driver genes of EndMT in CMs have not been fully elucidated. Single-cell sequencing provides effective strategies to identify and probe cellular subpopulations and their internal characteristics^12^. We identified the EndMT cell type for the first time at the single-cell level. In our samples, 5676 EndMT cells were detected by canonical marker genes^46^ (Fig. 4a, b and Supplementary Fig. 8). This cell type was also confirmed in human CM samples by immunofluorescence staining of CD31 and a-SMA (Fig. 4c).

To obtain potential novel marker genes, we performed differential gene analysis comparing ECs, EndMT cells and fibroblasts, and eight potential marker genes of EndMT cells were identified: *RAMP2*, *IFI27*, *TM4SF1*, *GNG11*, *TIMP3*, *IGFBP7*, *ID3*, and *CTGF* (Supplementary Fig. 9a). To further investigate the potential transition of ECs to EndMT cells and then to fibroblasts, we utilized the reversed graph embedding technique from Monocle v.2^17^ and reconstructed an unsupervised cell trajectory. The pseudotime trajectory axis indicated that ECs could transdifferentiate into EndMT cells and then into fibroblasts (Fig. 4d). Pseudotemporal expression dynamics of specific representative genes also marked the progression of ECs to EndMT cells and then to fibroblasts (Fig. 4e).

EndMT, an intermediate transition state, was a predominantly immune-activated state characterized by pathways involved in the regulation of immune effector processes, chemokine production and leukocyte cell‒cell adhesion signaling (Fig. 4f). Cell‒cell communication analysis revealed that EndMT cells were more crucial contributors to immune-related pathways such as IL1 and CXCL signaling than ECs or fibroblasts (Fig. 4g).

To further explore the differences between CM lesion samples and control samples within EndMT cells, we analyzed their differences in gene expression in EndMT cells. The representative significant differentially expressed genes are shown in Fig. 4h. The GO pathway analysis and GSEA revealed an enrichment of the activation of immune responses in upregulated genes and the PERK-mediated unfolded protein response in downregulated genes, respectively (Fig. 4i and Supplementary Fig. 9b).

### SPI1 upregulation was critical to promote EndMT

**Fig. 5:**
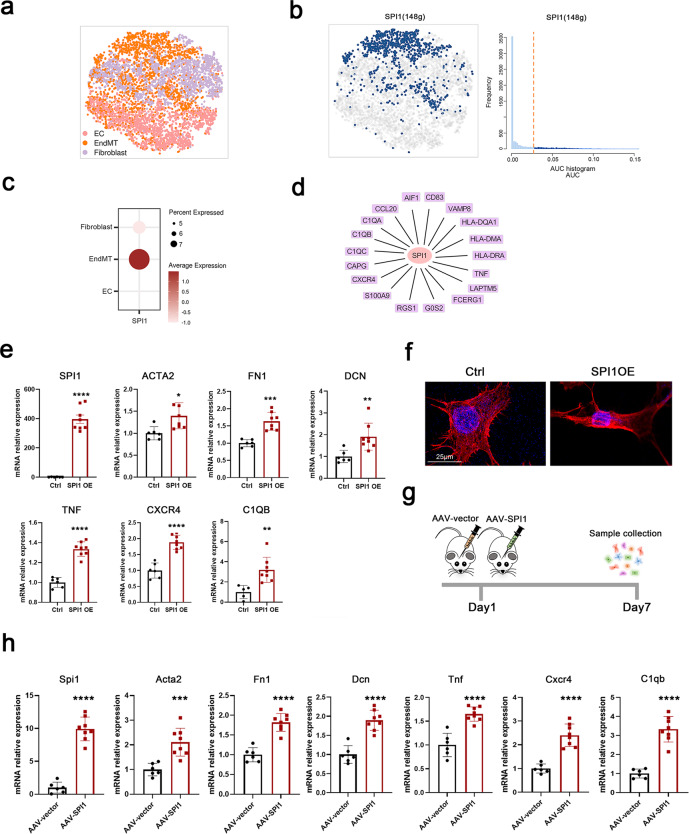
SPI1 plays a key role in EndMT. **a** SCENIC-based t-distributed stochastic neighbor embedding (tSNE) plot for the distributions of ECs, EndMT cells and fibroblasts. **b** SCENIC analysis predicted the transcription factor SPI1 as a specific hub (distinguished from ECs and fibroblasts) governing the EndMT cell state (left panel). SPI1 regulon activities were quantified using AUCell (right panel). **c** Dot plot for SPI1 expression in ECs, EndMT cells and fibroblasts. **d** Representative target genes of SPI1 overlapping with dynamic immune genes. **e** Expression levels of SPI1, EndMT markers (ACTA2, FN1 and DCN) and immune markers (TNF, CXCR4 and C1QB) regulated by SPI1 analyzed via real-time quantitative PCR (RT‒qPCR) assay in HUVECs after SPI1 and control plasmid transfection. **f** Morphological changes and cytoskeletal reorganization of HUVECs are shown by rhodamine-phalloidin staining (red) in the SPI1 overexpression and control groups. DAPI-stained nuclei are shown in blue. Scale bars, 25 µm. **g** SPI1 overexpression in the ECs of mice was induced by the injection of AAV-SPI1 with the TIE promoter stereotactically into the basal ganglia. Primary cerebrovascular ECs were collected 7 days after AAV-SPI1 and AAV-vector injection. **h** Expression levels of SPI1, EndMT markers (Acta2, Fn1 and Dcn) and immune markers (Tnf, Cxcr4 and C1qb) regulated by SPI1 analyzed via RT‒qPCR assay in primary ECs of cerebrovascular after AAV-SPI1 and AAV-vector injection. Primary ECs were isolated following the standard procedure of the MicroBeads kit and then used for RNA extraction and RT‒qPCR assay without culturing. Data are represented as the mean ± SD (AAV-vector group, *n* = 6; AAV-SPI1 group, *n* = 8). Statistics were performed using Student’s *t* test, and significance was determined as *P < 0.05, **P < 0.01, ***P < 0.001, ****P < 0.0001.

To identify the master regulators of EndMT cells, we constructed transcriptional regulatory networks with transcriptional regulators and their target genes by applying SCENIC analysis, including ECs, EndMT cells and fibroblasts (Fig. 5a). SPI1 was the potential transcription factor predicted to specifically govern the cell state of EndMT (Fig. 5b). In addition, SPI1 was mainly expressed in EndMT cells and displayed low expression levels in ECs and fibroblasts (Fig. 5c). The importance of SPI1 in inflammatory pathways has been well delineated in previous studies^47–50^, which matched the immune-activated feature of the EndMT cell type. Consistent with this, SPI1 occupied regulatory regions in a large proportion of genes involved in inflammatory pathways, such as *C1QA*, *C1QB*, *C1QC*, and *S100A9*, in our results (Fig. 5d), suggesting the reliable regulatory role of SPI1 in EndMT cells.

To validate the involvement of SPI1 in EndMT, we overexpressed *SPI1* in human umbilical vein endothelial cells (HUVECs) by plasmid transfection. Reverse transcription quantitative PCR (RT‒qPCR) analysis revealed that *SPI1* expression was markedly elevated after transfection. Simultaneously, the mesenchymal cell markers *ACTA2*, *FN1*, and *DCN* were increased after *SPI1* overexpression, and phalloidin staining showed that SPI1 promoted the transformation of HUVECs from a cobblestone-like epithelial phenotype to a spindle-like mesenchymal phenotype, which demonstrated that SPI1 promoted the EndMT process in HUVECs (Fig. 5e, f). In addition, SPI1 triggered immune activation in HUVECs that upregulated the mRNA levels of multiple immune factors, such as *TNF*, *CXCR4*, and *C1QB* (Fig. 5e). We then stereotactically injected AAV9-TIE-SPI1 into the brains of the mice to induce Spi1 overexpression in vivo (Fig. 5g). Seven days after injection, although no striking phenotypes were observed, when brain primary ECs were separated (Supplementary Fig. 10a) for RNA extraction and RT‒qPCR assay, the expression levels of Spi1, mesenchymal cell markers, and immune factors were all markedly upregulated, which verified the function of Spi1 in facilitating EndMT at the animal level in vivo (Fig. 5h).

### A specific fibroblast-like phenotype was identified in the SMCs of CM lesions

**Fig. 6:**
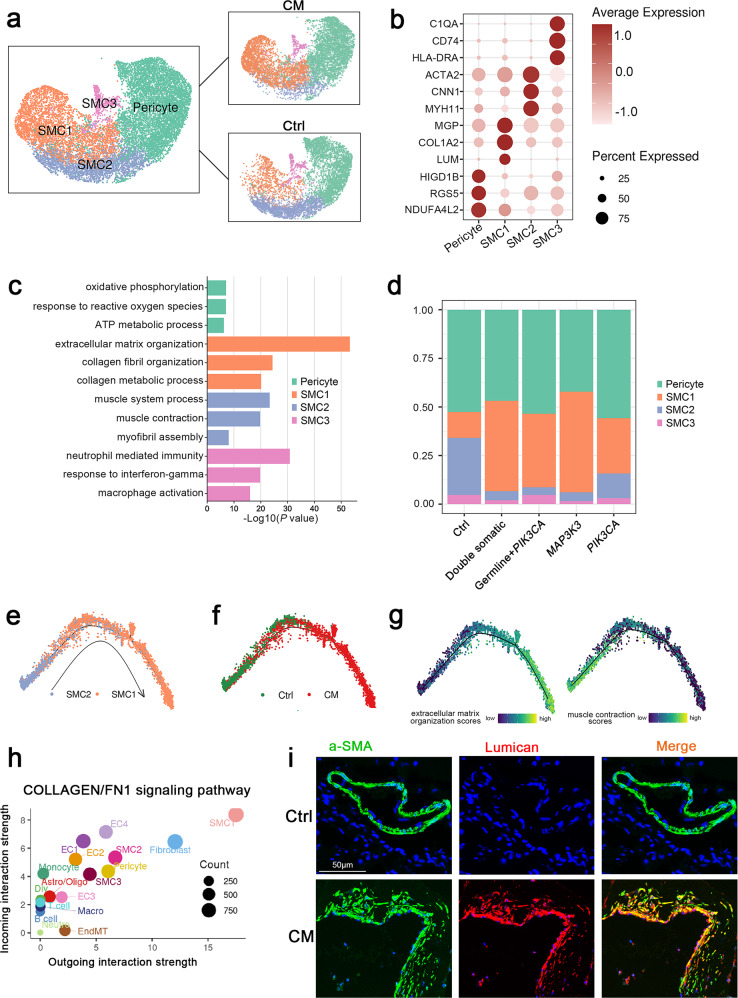
Mural cell clusters. **a** UMAP plot for the distributions of four mural cell subclusters (left panel) divided by control and CM groups (right panel). **b** Marker genes of four mural cell subclusters. **c** GO enrichment analysis of DEGs in four mural cell subclusters. **d** The proportions of the four mural cell subclusters in the different groups. Monocle 2 pseudotime analysis for SMC1 and SMC2 marked with cell types (**e**), control and CMs groups (**f**). **g** Extracellular matrix organization and muscle contraction scores along the pseudotime axis. Specific genes are shown in Supplementary Table 4. **h** Scatter plot showing outgoing (*x*-axis) and incoming (*y*-axis) cellular interaction strength of COLLAGEN and FN1 signaling pathways in all cell types. **i** Immunofluorescence staining of SMC1 marked by a-SMA and lumican in (**b**) in the control and CM groups. Lumican, red; a-SMA, green and DAPI-stained nuclei shown in blue. Scale bars, 50 µm.

Mural cells are essential components of blood vessels and crucial for normal development and homeostasis^51^. To investigate the transcriptional heterogeneity of mural cells in CMs, we selected and reclustered them into four subpopulations: pericytes and three types of SMCs, which were defined by typical marker genes and their cellular functions (Fig. 6a and Supplementary Fig. 11).

Differential expression and GO enrichment analysis indicated that pericytes marked with *HIGD1B*, *RGS5* and *NDUFA4L2*^22^ were associated with oxidative phosphorylation, response to reactive oxygen species, and ATP metabolic processes; SMC1 highly expressed fibroblast marker genes such as *MGP*, *COL1A2* and *LUM* and was enriched in extracellular matrix related pathways, thus, these SMCs were defined as fibroblast-like SMCs; SMC2 presented as typical SMCs, marked by *ACTA2*, *CNN1* and *MYH11* expression, and was related to muscle system processes, muscle contraction, and myofibril assembly. Immune genes (such as *C1QA*, *CD74* and *HLA-DRA*) were specifically expressed in SMC4; therefore, we defined these SMCs as immune-related SMCs, and functional analysis also confirmed the immunological characteristics of SMC4 (Fig. 6b, c).

Subsequently, we calculated the proportions of four mural subclusters in different groups. Notably, SMC1 had a higher proportion, while SMC2 had a lower proportion in all CM lesion groups (Fig. 6d and Supplementary Figs. 12–13). Given the cellular characteristics and proportions of the two SMC subclusters, we speculated that SMCs underwent more phenotypic switching in CM lesions, acquiring a specific fibroblast-like phenotype^52^. Next, trajectory analysis derived from Monocle confirmed the potential transition: typical SMCs gradually transdifferentiated into fibroblast-like SMCs along the pseudotime axis. (Fig. 6e) Correspondingly, SMCs in lesions also tended to aggregate along the trajectory (Fig. 6f). Pseudotemporal expression of dynamic genes related to extracellular matrix organization and muscle contraction also marked the progression of SMC phenotypic switching (Fig. 6g). Cellular interaction analysis identified SMC1 as the strongest contributor to the COLLAGEN and FN1 signaling pathways in all cell types (Fig. 6h). SMC1 was also confirmed in human CM samples by immunofluorescence staining of a-SMA and lumican (Fig. 6i).

### TWIST1 induced SMC phenotypic switching

**Fig. 7:**
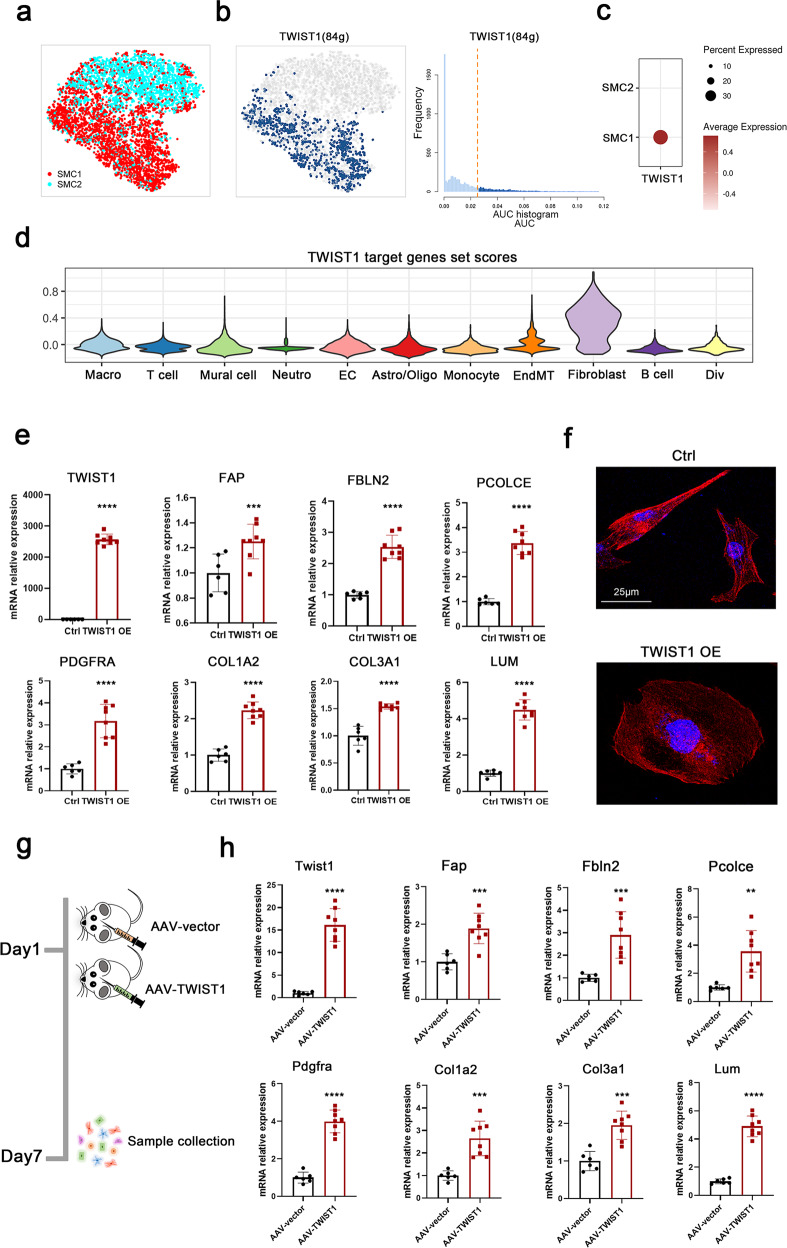
TWIST1 induced phenotypic switching of SMCs. **a** SCENIC-based tSNE plot for the distributions of SMC1 and SMC2. **b** SCENIC analysis predicted the transcription factor TWIST1 as a specific hub distinguished from SMC2 governing SMC1 cell states (left panel). TWIST1 regulon activities were quantified using AUCell (right panel). **c** Dot plot for TWIST1 expression in SMC1 and SMC2. **d** TWIST1 target gene set scores in 11 cell types. Specific genes are shown in Supplementary Table 5. **e** Expression levels of TWIST1 and SMC phenotypic switching markers analyzed via RT‒qPCR assay in HASMCs after TWIST1 and control plasmid transfection. **f** Morphological changes and cytoskeletal reorganization of HASMCs are shown by rhodamine-phalloidin staining (red) in the TWIST1 overexpression and control groups. DAPI-stained nuclei are shown in blue. Scale bars, 25 µm. **g** TWIST1 overexpression in SMCs of mice was induced by stereotaxic injection of AAV-SPI1 with the SM22a promoter into the basal ganglia. Primary cerebrovascular SMCs were collected 7 days after AAV-TWIST1 and AAV-vector injection. **h** Expression levels of Twist1 and SMC phenotypic switching markers analyzed via RT‒qPCR assay in primary SMCs of cerebrovascular after AAV-TWIST1 and AAV-vector injection. Primary SMCs were isolated following the standard procedure of the MicroBeads kit and then used for RNA extraction and RT‒qPCR assay without culturing. Data are represented as the mean ± SD (AAV-vector group, *n* = 6; AAV-TWIST1 group, *n* = 8). Statistics were performed using Student’s *t* test, and significance was determined as **P < 0.01, ***P < 0.001, ****P < 0.0001.

We then employed SCENIC analysis to identify the regulators of fibroblast-like SMCs. The transcription factor TWIST1 was predicted to specifically govern the cell states of fibroblast-like SMCs distinct from those of typical SMCs (Fig. 7a, b). Simultaneously, TWIST1 was highly expressed in SMC1 and expressed at lower levels in SMC2. (Fig. 7c) The target genes of TWIST1 also had fibroblast expression specificity, again indicating the fibroblast-like features of SMC1 (Fig. 7d).

To verify the prediction, we transfected the TWIST1 plasmid into human arterial smooth muscle cells (HASMCs). RT‒qPCR analysis revealed that TWIST1 expression was markedly increased after transfection, and the mRNA levels of TWIST1 target genes such as *FAP*, *FBLN2*, *PCOLCE* and *PDGFRA* and typical fibroblast markers such as *COL1A2*, *COL3A1* and *LUM* were also significantly upregulated after *TWIST1* overexpression (Fig. 7e). We also observed significant morphological changes in spindle-like HASMCs after TWIST1 overexpression by phalloidin staining (Fig. 7f). We then stereotactically injected AAV9-SM22a-TWIST1 into the brains of mice to induce TWIST1 overexpression in SMCs in vivo (Fig. 7g). Seven days after injection, although we did not observe striking phenotypes, we separated brain primary SMCs (Supplementary Fig. 10b) and used them for RNA extraction and RT‒qPCR assays. Strikingly, the expression levels of Twist1, Twist1 target genes and typical fibroblast markers were all significantly upregulated in primary SMCs (Fig. 7h). The above results showed that TWIST1 could induce SMC phenotypic switching in vitro and in vivo.

## Discussion

**Fig. 8:**
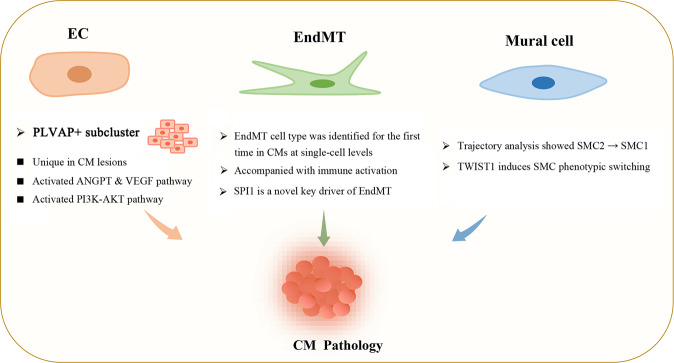
Endothelial cells, EndMT cells and mural cells contribute to the pathogenesis of cavernous malformations. A new EC subcluster marked by PLVAP expression with activating ANGPT and VEGF and PI3K-AKT pathway is unique in CM lesions. EndMT cells accompanied by strong immune activation were identified for the first time in CMs at the single-cell level. SPI1 is a novel key driver of EndMT. Trajectory analysis showed that typical SMCs (SMC2) gradually transdifferentiated into fibroblast-like SMCs (SMC1) in CM lesions. TWIST1 could induce SMC phenotypic switching.

In this study, we analyzed 112,670 cells after quality control. To our knowledge, this is the largest single-cell sequencing dataset for CMs thus far^1,13^. Here, we present a comprehensive cellular composition of CMs and provide novel insight into how the gene expression landscape is altered in CMs, especially in ECs, EndMT cells and mural cells (Fig. 8). Our analyses provide a blueprint for interrogating the cellular and molecular basis of CMs and will fuel advances in CM clinical therapy.

ECs have long been suggested to represent a heterogeneous population^53^, but the extent of heterogeneity in CM patients has hitherto remained unexplored. Fabrizio Orsenigo et al. characterized subclasses of brain ECs using a mouse model^13^. Here, we have shown that the ECs of CM lesion and control samples represented a heterogeneous population and were distributed across four different clusters on the basis of their gene expression. Among them, we identified a subcluster with a high level of *MFSD2A* expression. MFSD2A, a key regulator of BBB function, is required to suppress endothelial transcytosis in the CNS. Moreover, Mfsd2a is uniquely required for normal brain growth^54^. Genetic ablation of Mfsd2a results in a leaky BBB from embryonic stages through adulthood in a mouse model^55^. At present, the highest proportion of ECs in control group samples was also observed in this subcluster. Hence, these data allowed us to conclude that the EC2 subcluster exhibited a higher expression level of *MFSD2A* and normal EC functions.

Remarkably, we found a unique subcluster in CM lesion samples with a high expression level of *PLVAP* that was almost nonexistent in control samples. *PLVAP* encodes an endothelial-specific type II integral membrane protein that forms homodimers thought to create radial fibrils/spokes in the ring-and-spoke structure of the diaphragms of endothelial fenestrae, transendothelial channels, and caveolae^56^. In addition, Plvap is involved in endothelial vesicle trafficking, is highly expressed in permeable peripheral vessels and is upregulated in CNS endothelial cells during pathological breakdown of the BBB^32^. Thus, PLVAP-positive EC subcluster 4 exhibited increased permeability under pathological conditions, which matched the features of BBB impairment in CCMs. EC4 marker genes, including *Plvap*, *F2rl3* and *Igfbp5*, were also upregulated in *Pdcd10*-KO mice compared to wild-type mice^13^. We further confirmed the presence of PLVAP-positive EC subcluster 4 in CM lesion samples by immunofluorescence staining. This report is thus the first to identify a unique EC subcluster that distinguishes CM lesion samples from control samples, which may pave the way for dissecting EC heterogeneity in CMs.

EndMT has been described in different pathologies, and it is defined as the acquisition of mesenchymal and stem-cell-like characteristics by the endothelium^45^. Nevertheless, Fabrizio et al. did not identify this cell type in their single-cell dataset for a *Pdcd10*-KO mouse model, which could be due to sample and cell count limitations^13^. Remarkably, by focusing on a larger number of CM samples, we identified the EndMT cell type of CMs for the first time at the single-cell level and provided a novel set of potential marker genes for EndMT cell annotation. Pseudotemporal ordering demonstrated that ECs transdifferentiate into EndMT cells and then into fibroblasts. EndMT, an intermediate transition state, was a predominantly immune-activated state. EndMT can be induced by proinflammatory cytokines in TGF-β-dependent or TGF-β-independent manners and relies on the induction of Snail by NF-κB^57^. Intriguingly, SPI1 was predicted to be the most promising transcription factor for EndMT in our study. SPI1 encodes an ETS-domain transcription factor and participates in immune responses^58,59^; SPI1 has never been reported to be related to EndMT. We overexpressed SPI1 in HUVECs and the brain primary ECs of mice and found that they acquired mesenchymal characteristics, which confirmed that the progression of EndMT was induced by SPI1. We also found that some representative immune factors showed elevated expression after SPI1 overexpression, which verified the immune features during the EndMT state.

SMCs possess remarkable phenotypic plasticity that allows rapid adaptation to fluctuating environmental cues, including during the development and progression of vascular diseases such as atherosclerosis, aortic dissection and aneurysm^52,60,61^. In this study, we first identified fibroblast-like SMCs in CM lesions. Cellular trajectory analysis indicated that SMCs gradually lost muscle contraction features and acquired extracellular matrix characteristics in the progression from normal tissue to CM lesions. TWIST1, a basic helix-loop-helix protein involved in multiple physiological and pathological processes^62^, was predicted to be the critical transcription factor governing fibroblast-like SMCs. The overexpression of TWIST1 in HASMCs and the brain primary SMCs of mice resulted in the upregulation of typical fibroblast markers, indicating the critical role of TWIST1 in SMC phenotypic switching and hinting that this could be a promising target for vascular remodeling and repair in CMs.

Although no specific CM phenotype was observed in mice, the in vivo experiments confirmed that EC-specific or SMC-specific overexpression had effects in vivo. We speculated that their expression was probably insufficient to cause the CM phenotype. Additional in vitro and in vivo studies are needed to determine whether *SPI1* and *TWIST1* overexpression is the cause or the effect of CMs.

In summary, we constructed a comprehensive single-cell transcriptome atlas from 12 CM samples and 3 control samples. A new EC subpopulation marked by PLVAP expression was uniquely identified in lesions. The marker genes of this EC subpopulation were also upregulated in the ECs of *Pdcd10*-KO mice. Endothelial-to-mesenchymal transition (EndMT) cells were identified for the first time in CMs at the single-cell level, and these cells were accompanied by strong immune activation. The transcription factor SPI1 was predicted and verified to be a novel key driver of EndMT. A specific fibroblast-like phenotype was more prevalent in lesion SMCs, suggesting a new mechanism for vessel reconstruction and repair in CMs, and we found that TWIST1 could induce SMC phenotypic switching. Overall, our findings provide a comprehensive transcriptomic landscape of human CMs at single-cell resolution and present potential therapeutic targets for CMs.

## Supplementary information

Supplementary materials
