# Supplementary material for: Single-cell sequencing reveals that endothelial cells, EndMT cells and mural cells contribute to the pathogenesis of cavernous malformations
Source: Exp Mol Med. 2023 Mar 13;55(3):628–42. doi: 10.1038/s12276-023-00962-w (PMC10073145; doi:10.1038/s12276-023-00962-w)
Supplement: Supplementary file 1 — Supplementary materials [file 12276_2023_962_MOESM1_ESM.pdf]

Supplementary materials

Supplementary Tables

Supplementary Table 1: Maker genes of eleven cell types.

Clusters	Markers
Macro	*C1QA C1QB*
T cell	*CD3D CD3E*
Mural cell	*ACTA2 MYH11*
Neutro	*S100A8 S100A9*
EC	*PECAM1 VWF*
Astro/Oligo	*GFAP ALDH1L1 CNP MOG*
EndMT	*ACTA2 FN1 PECAM1 LUM*
Monocyte	*FCN CD14*
fibroblast	*LUM COL1A2 DCN*
B cell	*CD79A MS4A1*
Div	*MKI67 TOP2A C1QA CD3D*

Supplementary Table 2: Human primer sequences for real-time quantitative PCR.

Gene	Primer sequence
ACTB-F	5'-CATGTACGTTGCTATCCAGGC-3'
ACTB-R	5'-CTCCTTAATGTCACGCACGAT-3'
SPI1-F	5'-GTGCCCTATGACACGGATCTA-3'
SPI1-R	5'-AGTCCCAGTAATGGTCGCTAT-3'
ACTA2-F	5'-CGGTGCTGTCTCTCTATGCC-3'
ACTA2-R	5'-CGCTCAGTCAGGATCTTCA-3'
FN1-F	5'-AGGAAGCCGAGGTTTTAACTG-3'
FN1-R	5'-AGGACGCTCATAAGTGTCACC-3'
DCN-F	5'-ATGAAGGCCACTATCATCCTCC-3'
DCN-R	5'-GTCGCGGTCATCAGGAACTT-3'
TNF-F	5'-GAGGCCAAGCCCTGGTATG-3'
TNF-R	5'-CGGGCCGATTGATCTCAGC-3'
CXCR4-F	5'-ACTACACCGAGGAAATGGGCT-3'
CXCR4-R	5'-CCCACAATGCCAGTTAAGAAGA-3'
C1QB-F	5'-AGGTGAATCGGGAGACTACAA-3'
C1QB-R	5'-CACTGCGGGGCTCATAATTG-3'
TWIST1-F	5'-GTCCGCAGTCTTACGAGGAG-3'
TWIST1-R	5'-GCTTGAGGGTCTGAATCTTGCT-3'
FAP-F	5'-ATGAGCTTCCTCGTCCAATTCA-3'
FAP-R	5'-AGACCACCAGAGAGCATATTTTG-3'
FBLN2-F	5'-CAGGTGGCCTCTAACACCATC-3'
FBLN2-R	5'-CTGCTTGCAGGGTCCATTGT-3'
PCOLCE-F	5'-TCTCATTCCGAGTCTTCGACC-3'
PCOLCE-R	5'-GTCCCACAAAAGCGTCCGA-3'
PDGFRA-F	5'-TGGCAGTACCCCATGTCTGAA-3'
PDGFRA-R	5'-CCAAGACCGTCACAAAAAGGC-3'
LUM-F	5'-TAACTGCCCTGAAAGCTACCC-3'
LUM-R	5'-GGAGGCACCATTGGTACACTT-3'
COL1A2-F	5'-GAGCGGTAACAAGGGTGAGC-3'
COL1A2-R	5'-CTTCCCCATTAGGGCCTCTC-3'
COL3A1-F	5'-GGAGCTGGCTACTTCTCGC-3'
COL3A1-R	5'-GGGAACATCCTCCTTCAACAG-3'

Supplementary Table 3: Mouse primer sequences for real-time quantitative PCR.

Gene	Primer sequence
Actb-F	5'-GGCTGTATTCCCCTCCATCG-3'
Actb-R	5'-CCAGTTGGTAACAATGCCATGT-3'
Spi1-F	5'-TAGCGATCACTACTGGGATTTC-3'
Spi1-R	5'-GTTTGATAAGGGAAGCACATCC-3'
Acta2-F	5'-CCCAGACATCAGGGAGTAATGG-3'
Acta2-R	5'-TCTATCGGATACTTCAGCGTCA-3'
Fn1-F	5'-ATGTGGACCCCTCCTGATAGT-3'
Fn1-R	5'-GCCCAGTGATTTCAGCAAAGG-3'
Dcn-F	5'-CCCACTGAAAAACTCTGGGATT-3'
Dcn-R	5'-GGGATCGCAGTTATGTTGGTG-3'
Tnf-F	5'-CTGAACTTCGGGGTGATCGG-3'
Tnf-R	5'-GGCTTGTCACTCGAATTTTGAGA-3'
Cxcr4-F	5'-GACTGGCATAGTCGGCAATG-3'
Cxcr4-R	5'-AGAAGGGGAGTGTGATGACAAA-3'
C1qb-F	5'-CGTCGGCCCTAAGGGTACT-3'
C1qb-R	5'-GGGGCTGTTGATGGTCCTC-3'
Twist1-F	5'-GGACAAGCTGAGCAAGATTCA-3'
Twist1-R	5'-CGGAGAAGGCGTAGCTGAG-3'
Fap-F	5'-GTCACCTGATCGGCAATTTGT-3'
Fap-R	5'-TCGTAGATGTAGTATGTCGCTGT-3'
Fbln2-F	5'-AGTGGCCGTAAGTATGCTGC-3'
Fbln2-R	5'-GGAAGCTGGTAGCAAATGAGC-3'
Pcolce-F	5'-ACTACACGAGACCTGTGTTCC-3'
Pcolce-R	5'-GGCACCGTAATTGTCCAGATG-3'
Pdgfra-F	5'-ATGAGAGTGAGATCGAAGGCA-3'
Pdgfra-R	5'-CGGCAAGGTATGATGGCAGAG-3'
Lum-F	5'-CTCTTGCCTTGGCATTAGTCG-3'
Lum-R	5'-GGTCATCACAGTACATGGCAGT-3'
Col1a2-F	5'-AAGGGTGCTACTGGACTCCC-3'
Col1a2-R	5'-TTGTTACCGGATTCTCCTTTGG-3'
Col3a1-F	5'-CTGTAACATGGAAACTGGGGAAA-3'
Col3a1-R	5'-CCATAGCTGAACTGAAAACCACC-3'
Cd31-F	5'-CACAACAAACAAGCTAGCAAGA-3'
Cd31-R	5'-TTTGGCTGCAACTATTAAGGTG-3'
Cdh5-F	5'-CCCACTATCCGATACGAATACC-3'

Cdh5-R	5'-ATCCACATCTAGGACGTTGATG-3'
Pdgfrb-F	5'-GTCAAGATGCTGAAATCGACAG-3'
Pdgfrb-R	5'-GGGGTCCAAGATGACTCATAAT-3'

Supplementary Table 4: Gene sets of extracellular matrix organization, muscle contraction.

Terms	Gene sets
Extracellular matrix organization	*COL1A1 POSTN TIMP1 COL3A1 FN1 COL1A2 COL6A3 LUM MMP11 SERPINE1 VCAN CCDC80 SPARC COL5A1 SULF1 COL5A2 BGN COL6A1 COL6A2 LOXL2 COL4A1 COL4A2 FBN1 AEBP1 TGFBI TNC HTRA1 CTSK MMP2 FAP CST3 SERPINH1 COL8A1 LAMB1 TIMP2 LOX CYP1B1 COL12A1 FKBP10 PRDX4 MMP14 TNFRSF11B EMILIN1 ENG COL11A1 EFEMP2 COL15A1 LOXL1 ITGA11 FBLN5 LRP1 ADAMTS2 MMP23B B4GALT1 PXDN FBLN2 TNXB ADAM12 COL16A1 MFAP2 EXT1 FMOD CREB3L1 BMP1 MMP19 NID2 ADAMTS12 FBLN1 SULF2 RUNX1 CD44 ITGA5 VCAM1 ITGAV NID1 ITGB1 TGFB1 COLGALT1 TGFBR1 GAS6 FSCN1 PLOD3 SH3PXD2A QSOX1 DCN LAMA4 ITGB5 ITGA1 TGFB2 LTBP3 ANTXR1 CAPN2 LAMC1 HSPG2 THBS1 COL18A1 BMP2*
Muscle contraction	*DES MYH11 CNN1 PLN FLNA TPM2 MYL9 LMOD1 ACTG2 CALM1 ACTC1 MYL6 CRYAB PPP1R12B ACTA2 SLMAP SMTN SYNM TPM1 SORBS1 HSPB6 FXYD1 PGAM2 MYLK DMPK MYOM1 MYOCD VCL SNTA1 ATP1B1 PDE4D ATP2B4 TLN1 ATP2A2 ROCK1 MAP2K3*

Supplementary Table 5: TWIST1 target genes.

Terms	Gene sets
TWIST1 target genes	*COLEC12 CTSK DAB2 FAP FBLN2 FYN GFPT2 GXYLT2 HEY1 LEF1 MBOAT2 MFAP2 OLFML3 OSR2 PCOLCE PDGFRA RAB3IL1 SERPINF1 SFRP2 SOX9 ST3GAL4 STRA6*

Supplementary Figure

Supplementary Fig. 1: MRI characteristics, Integrative Genomic Viewer screen shot of WES and amplitude scatter plots of ddPCR.

Some of the patients have shown in our previous study[1] , here we showed the related information of new added patients.

**Group**	**Sample ID**	**The patient's numbers in our previous study**
Ctrl	Ctrl_1	/
	Ctrl_2	/
	Ctrl_3	/
Double somatic mutations	Double somatic_1	
	Double somatic_2	32
Germline mutations plus *PIK3CA* mutations	Germline+*PIK3CA*_1	
	Germline+*PIK3CA*_2	
MAP3K3 mutations	*MAP3K3*_1	
	*MAP3K3*_2	
	*MAP3K3*_3	*28*
PIK3CA mutations	*PIK3CA*_1	*31*
	*PIK3CA*_2	
	*PIK3CA*_3	*36*
	*PIK3CA*_4	
	*PIK3CA*_5	*35*

Patient Double_1

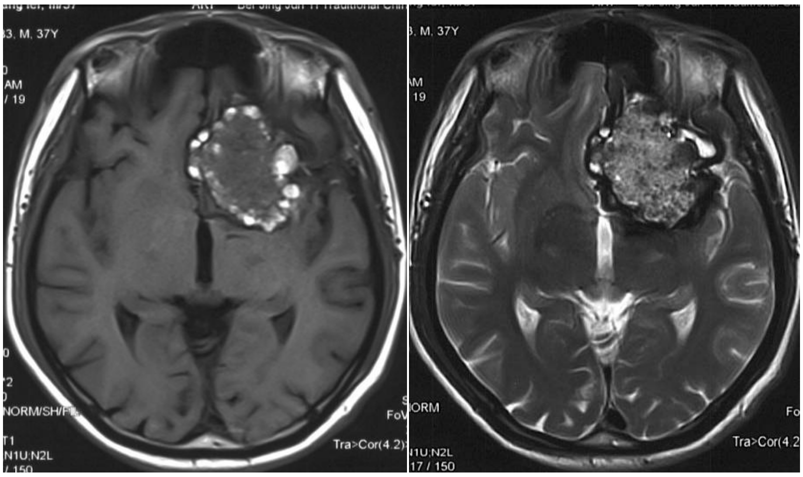

KRIT1 frameshift variant c.1671_1687del (p. L558Wfs*4)

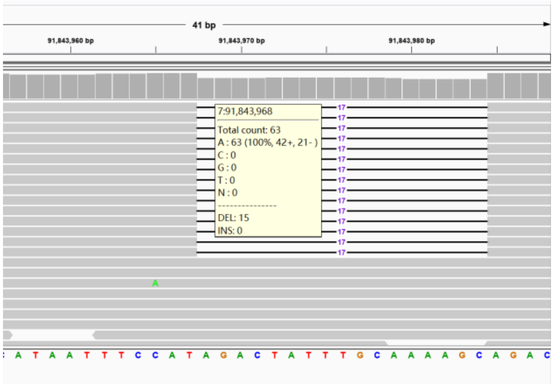

PIK3CA c.1633G>A(p.E545K)

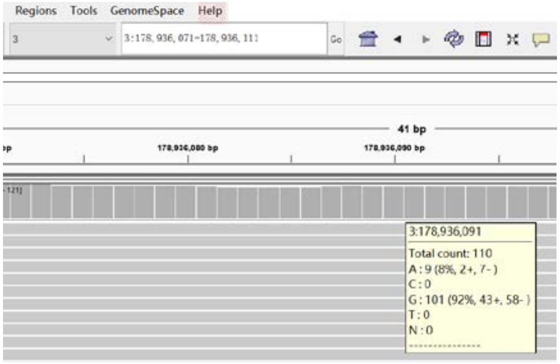

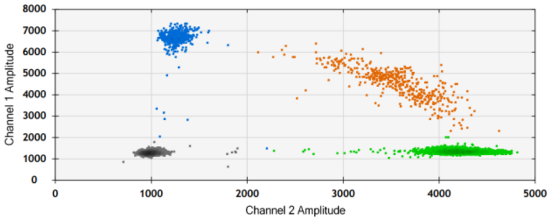

Patient Double_2

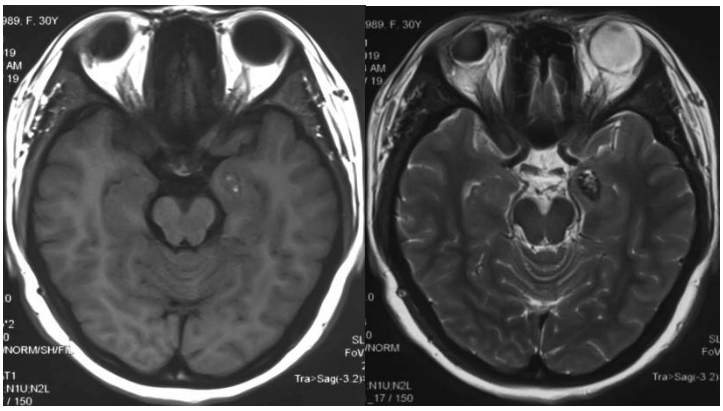

MAP3K3 c.1416C>G(p.I472M)

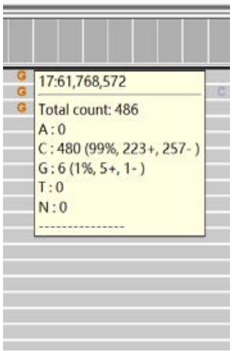

PIK3CA c.1633G>A(p.E545K)

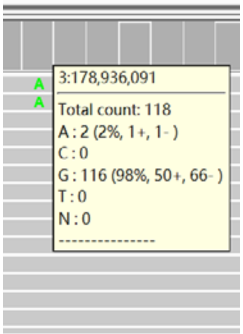

Patient Germline_1

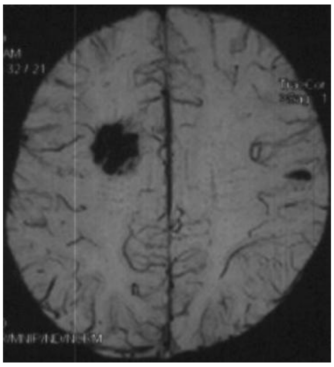

KRIT1 frameshift variant c.1961_1962del(p.K654Sfs*21)

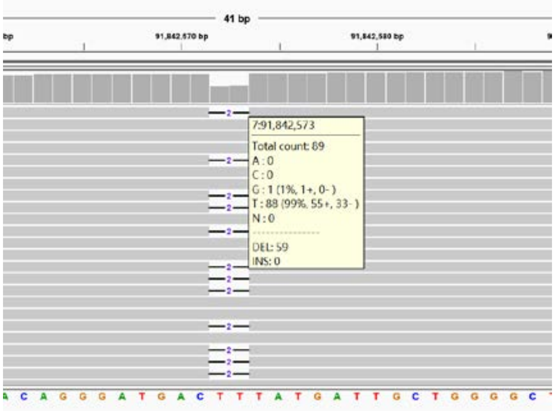

PIK3CA c.1624G>A(p.E542K)

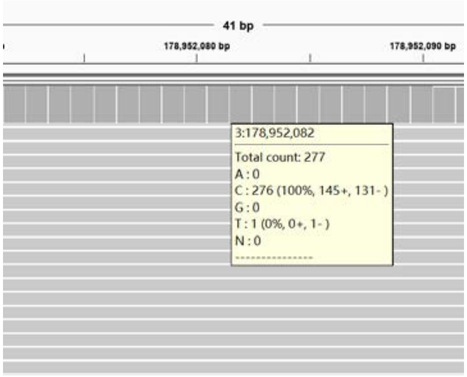

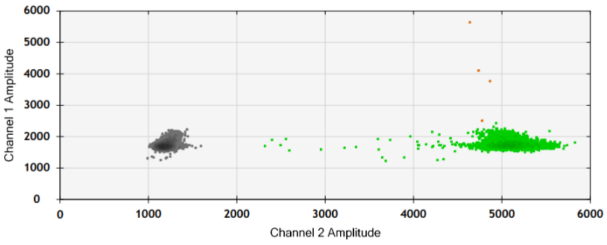

Patient Germline_2

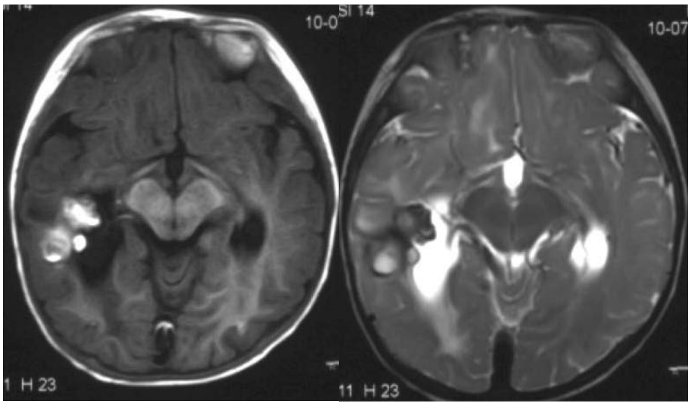

KRIT1 frameshift variant c.1649dup(p.L551Afs*17)

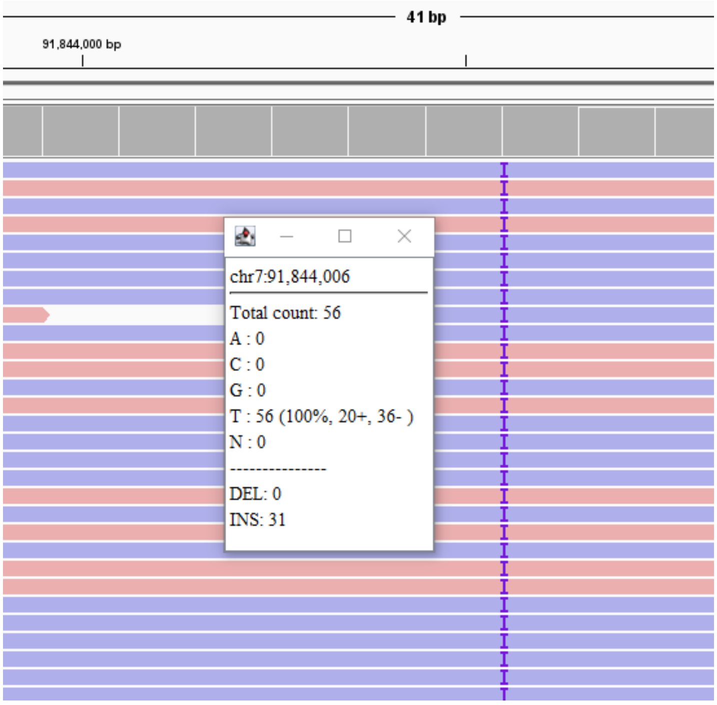

PIK3CA　　c.1624G>A(p.E542K)

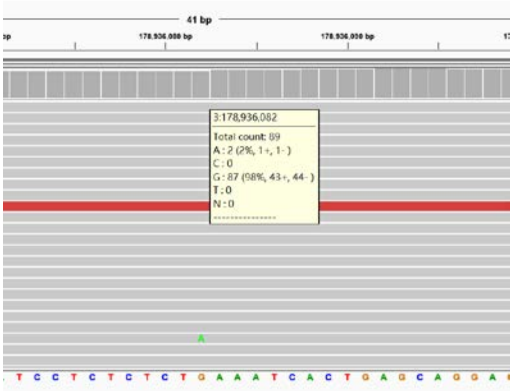

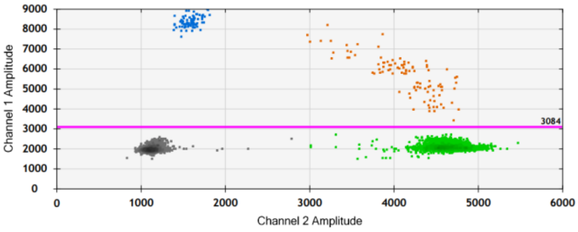

Patient *MAP3K3*_1

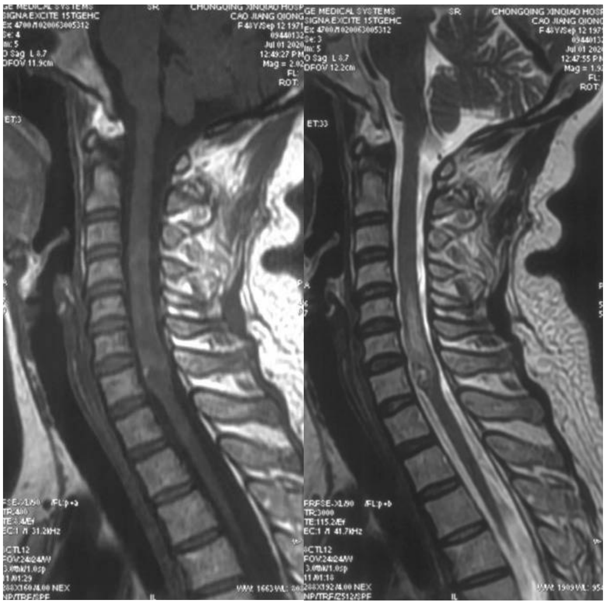

MAP3K3 c.1416C>G(p.I441M)

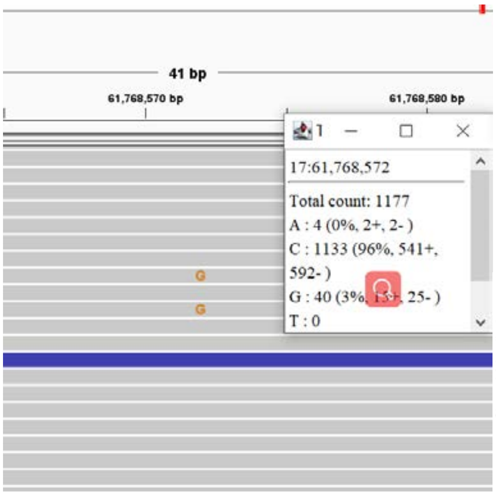

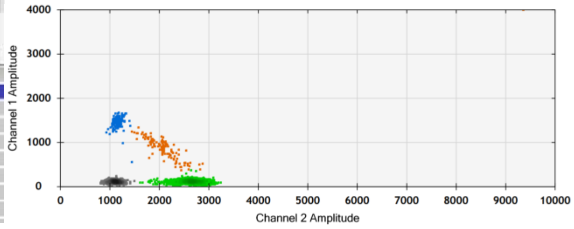

Patient *MAP3K3*_2

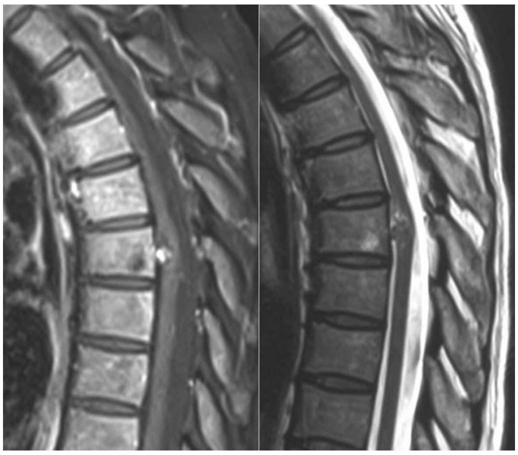

MAP3K3   c.1416C>G(p.I441M)

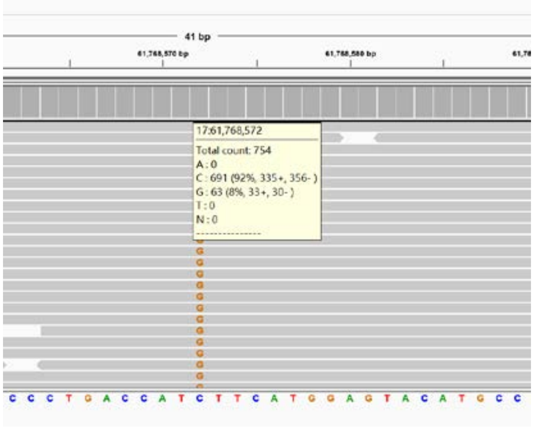

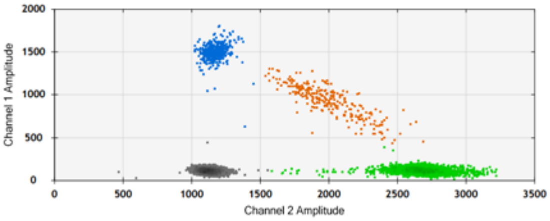

Patient *MAP3K3*_3

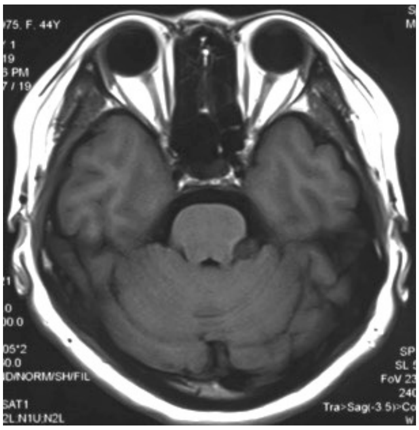

Patient 28

MAP3K3 c.1416C>G(p.I472M)

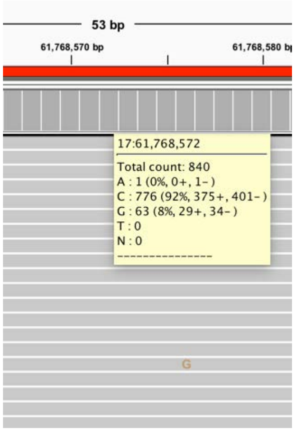

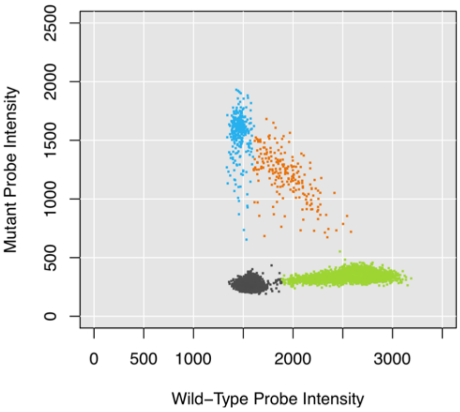

Patient *PIK3CA*_1

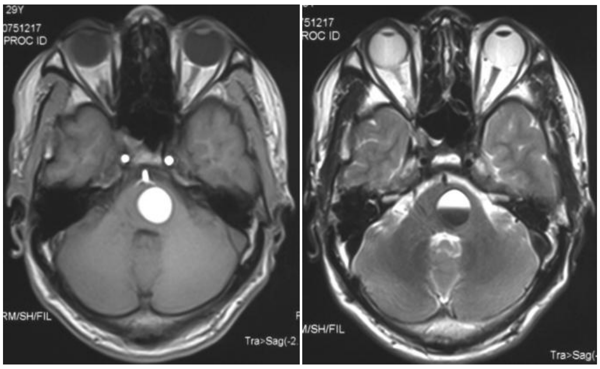

PIK3CA   c.1258T>C(p.C420R)

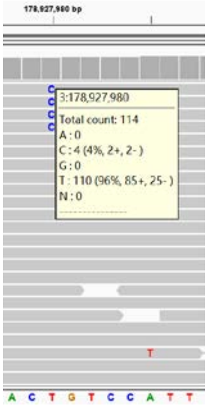

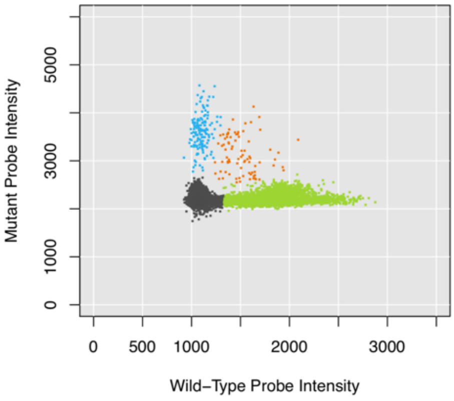

Patient *PIK3CA*_2

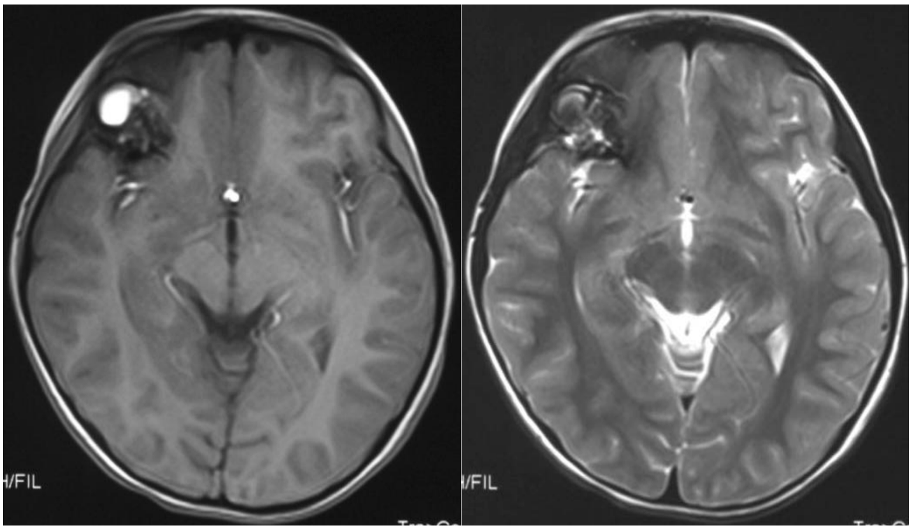

PIK3CA c.3140A>G(p.H1047R)

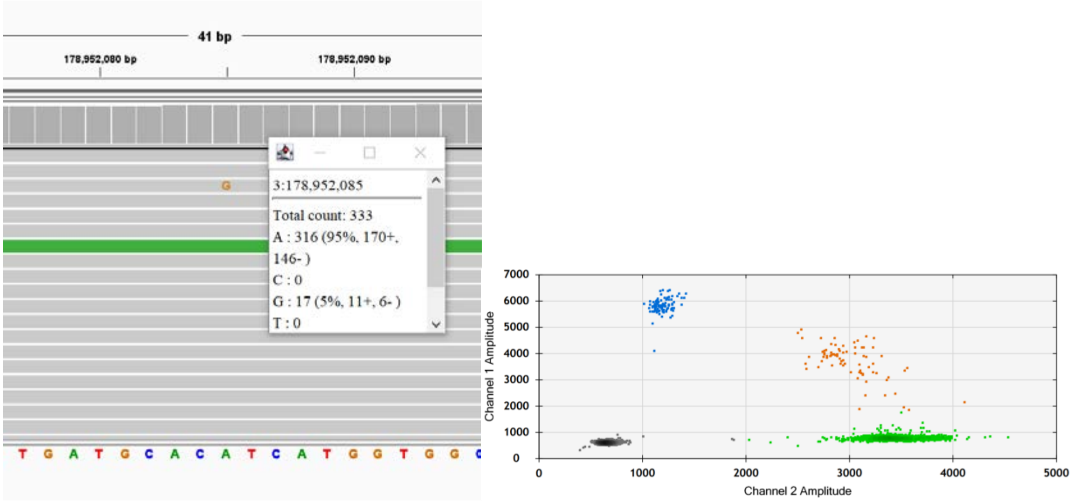

Patient *PIK3CA*_3

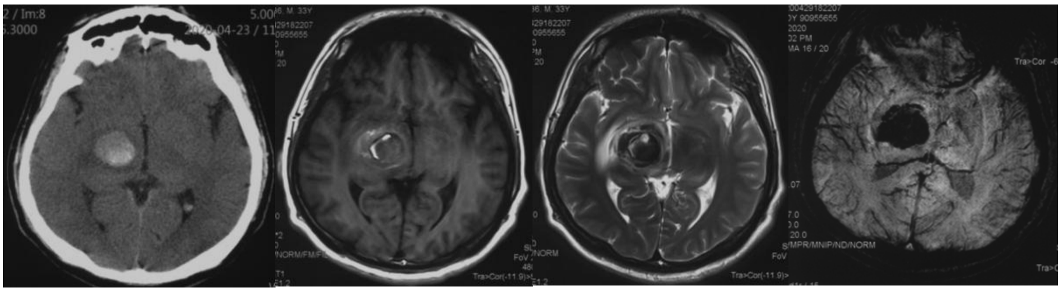

PIK3CA c.3140A>G(p.H1047R)

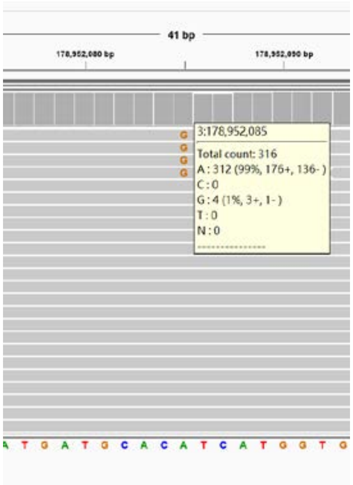

Patient *PIK3CA_4*

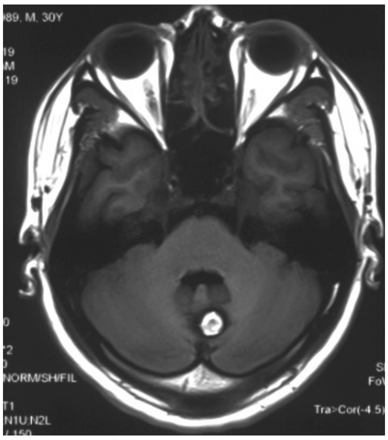

PIK3CA c.3140A>G(p.H1047R)

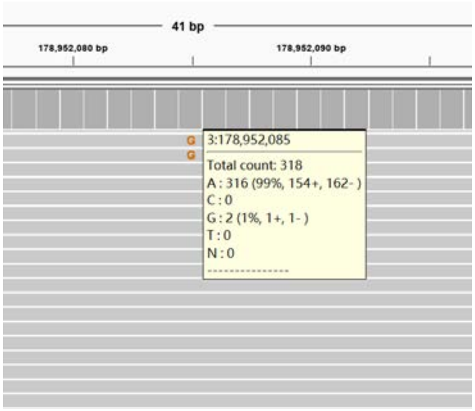

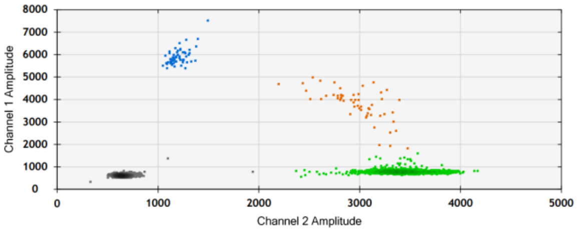

Patient *PIK3CA*_5

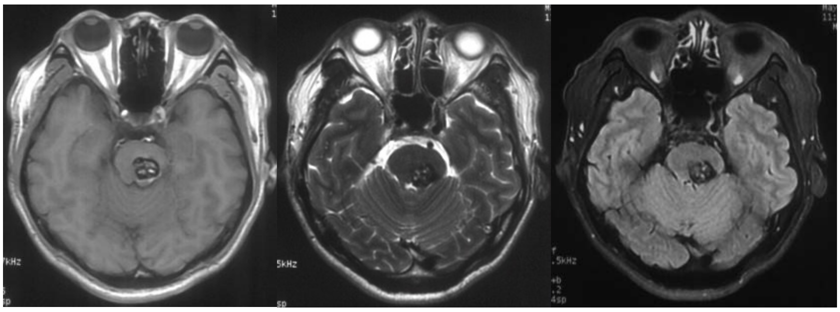

PIK3CA	c.3140A>G(p.H1047R)

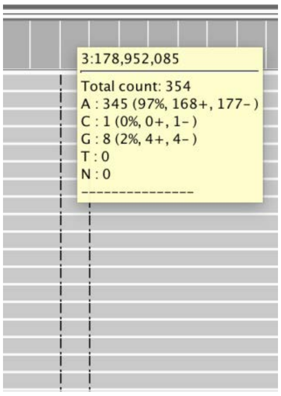

Supplementary Fig. 2. Landscape of eleven cell types in diffrenent groups.

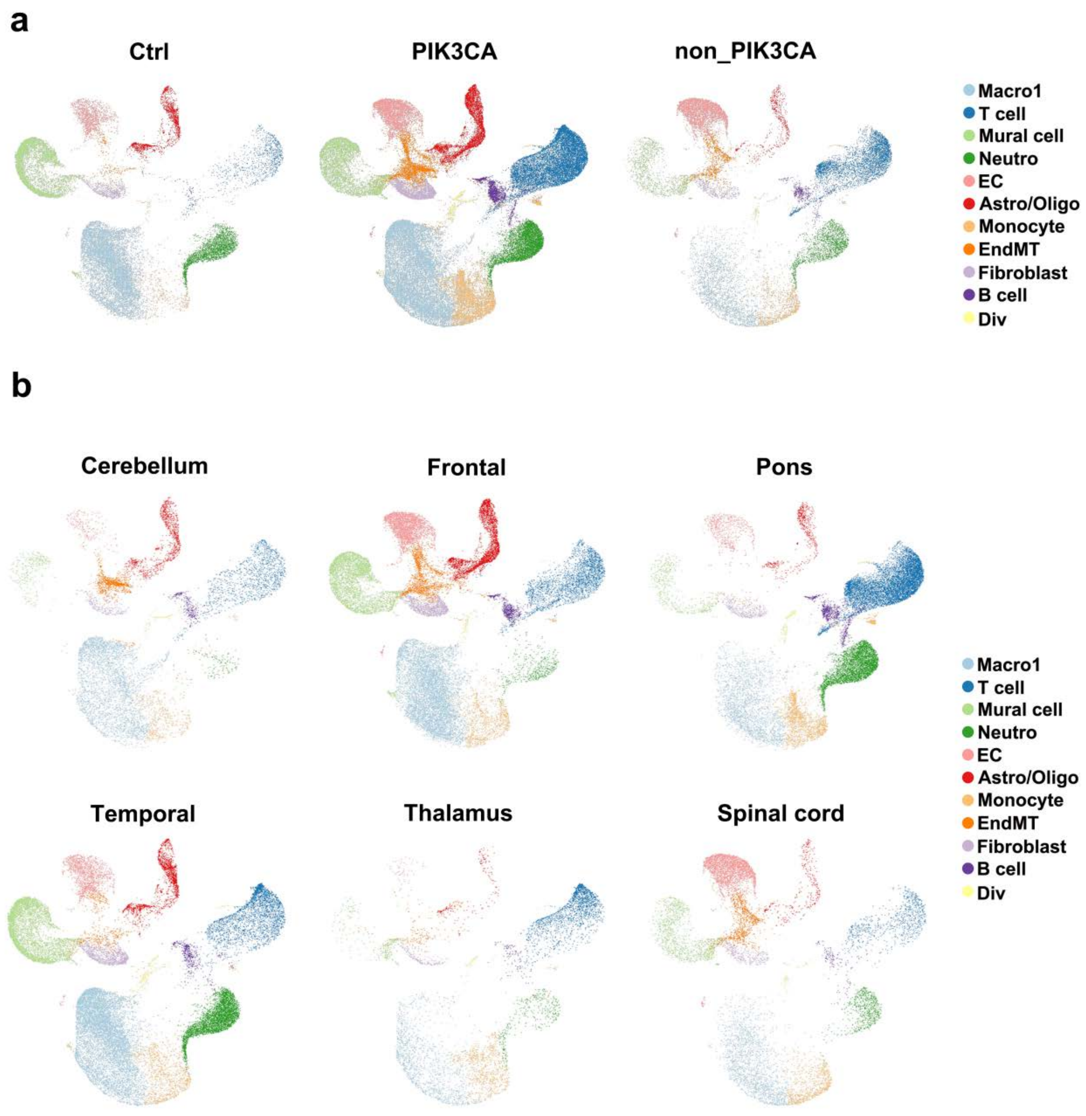

UMAP plots showed eleven cell types in (a) control, CMs with PIK3CA gene mutations and CMs without PIK3CA gene mutations groups; (b) different locations' groups. Macro, macrophage; Neutro, neutrophil; EC, endothelial cell; Astro/Oligo, astrocyte/oligodendrocyte; EndMT, endothelial-to-mesenchymal transition; Div, dividing immune cell.

Supplementary Fig. 3. Eleven cell type distributions of each sample shown with UMAP plots.

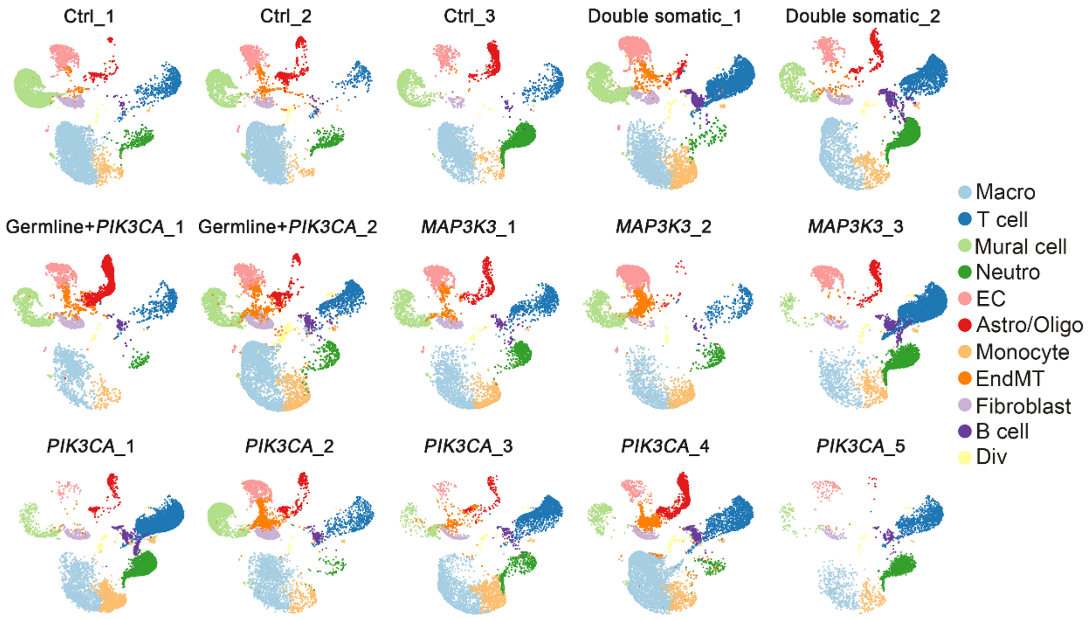

Macro, macrophage; Neutro, neutrophil; EC, endothelial cell; Astro/Oligo, astrocyte/oligodendrocyte; EndMT, endothelial-to-mesenchymal transition; Div, dividing immune cell.

Supplementary Fig. 4. UMAP plots for the number of genes and transcripts detected in each cell.

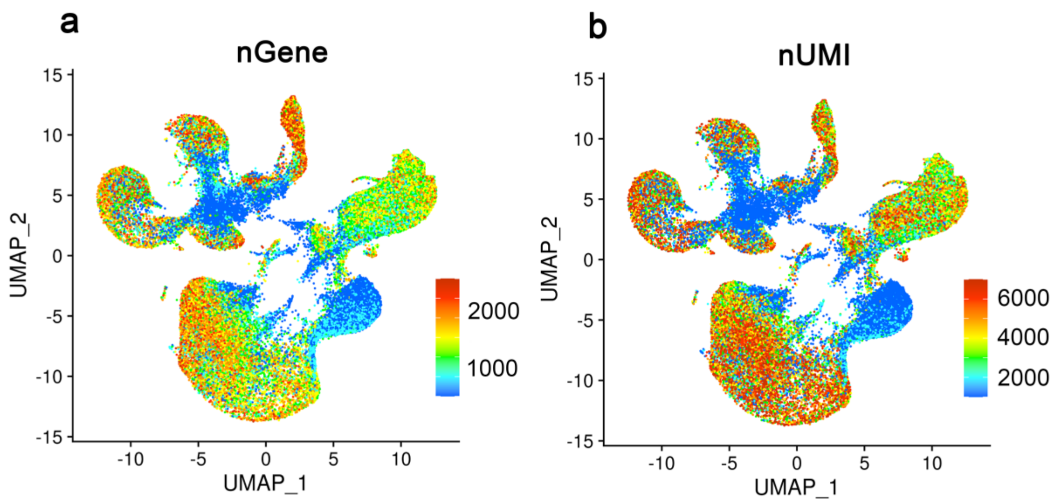

(a) number of genes (nGene) and (b) transcripts (nUMI).

Supplementary Fig. 5. Activated PI3K/AKT/mTOR and angiogenesis signaling pathways were observed in four mutation types of CM lesions, including CMs carrying with only *MAP3K3* mutation.

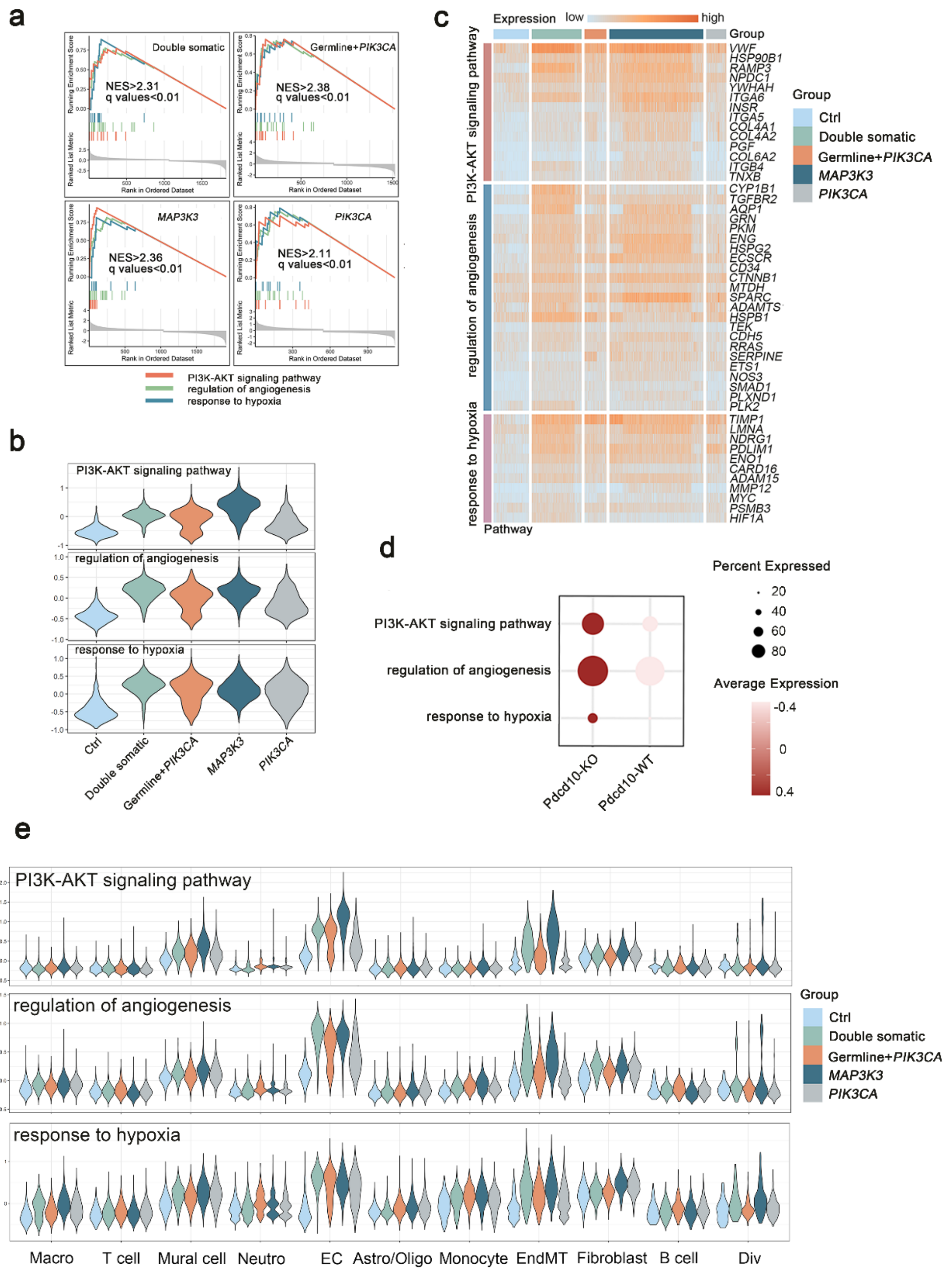

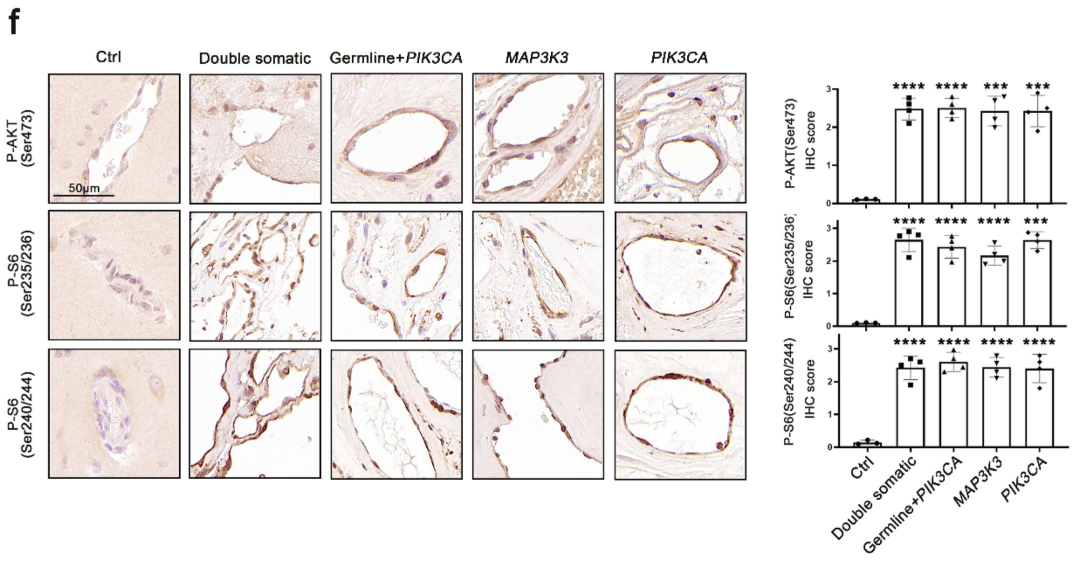

(a) GSEA and (b) gene set scores for the enrichment of PI3K-AKT signaling pathway, regulation of angiogenesis, and response to hypoxia in lesion ECs based on representative genes enriched in GO and KEGG analyses. (c) Heat map showing the expressions of representative genes enriched in above three pathways. Gene set scores of (d) above three pathways in brain ECs of *Pdcd10*-KO and *Pdcd10*-WT mice from public single-cell data (GSE155788)[2], and (e) PI3K-AKT signaling pathway, regulation of angiogenesis, and response to hypoxia in eleven cell types of different groups. (f) Immunohistochemical staining indicating the expressions of P-AKT (Ser473), P-S6 (Ser235/236) and P-S6 (Ser240/244) on different groups (left panel). Scale bars, 50 μm. IHC scores were determined by multiplying pathologist-assessed IHC intensity with fraction of positive cells (mean±SD) (right panel). No statistical differences between the four mutation groups (one-way ANOVA test), comparisons between each mutation group to control group (Student's t-test), ****P* < 0.001, *****P* < 0.0001. n=3-4. Macro, macrophage; Neutro, neutrophil; EC, endothelial cell; Astro/Oligo,

astrocyte/oligodendrocyte; EndMT, endothelial-to-mesenchymal transition; Div, dividing immune cell.

Supplementary Fig. 6. EC subclusters in different groups.

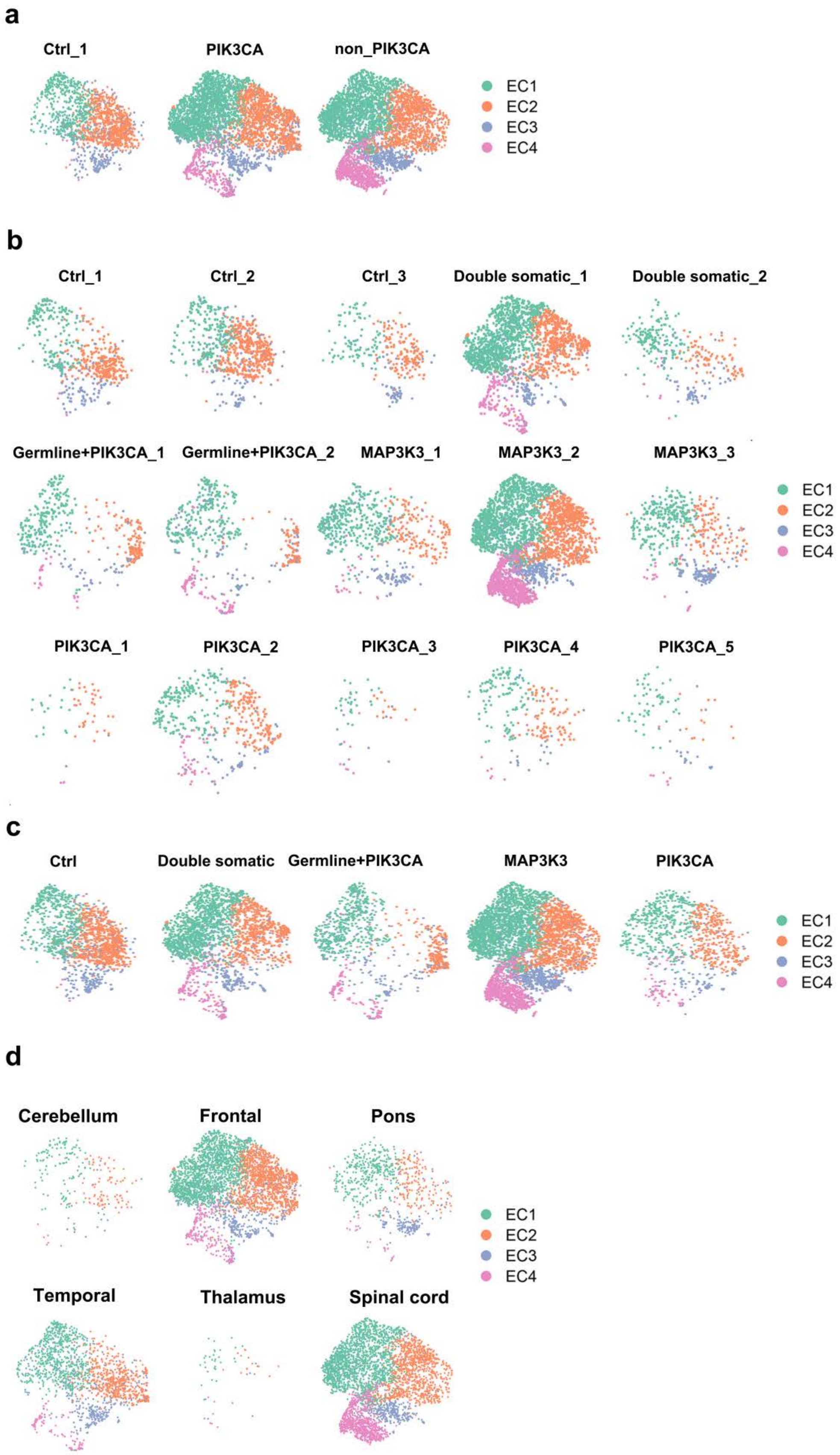

UMAP plots showed four EC subclusters in (a) control, CMs with PIK3CA gene mutations and CMs without PIK3CA gene mutations groups; (b) individual samples; (c) different genetic mutations' groups; (d) different laocations' groups.

Supplementary Fig. 7. Expression of predictive transcription factors in ECs.

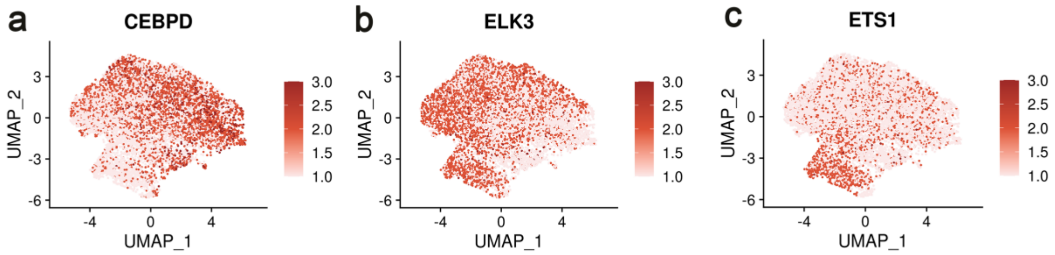

UMAP Plots for the distributions of (a) CEBPD, (b) ELK3, and (c) ETS1 in four EC subclusters.

Supplementary Fig. 8. UMAP plots displayed EndMT cell types divided into control, CMs with *PIK3CA* gene mutations and CMs without *PIK3CA* gene mutations groups.

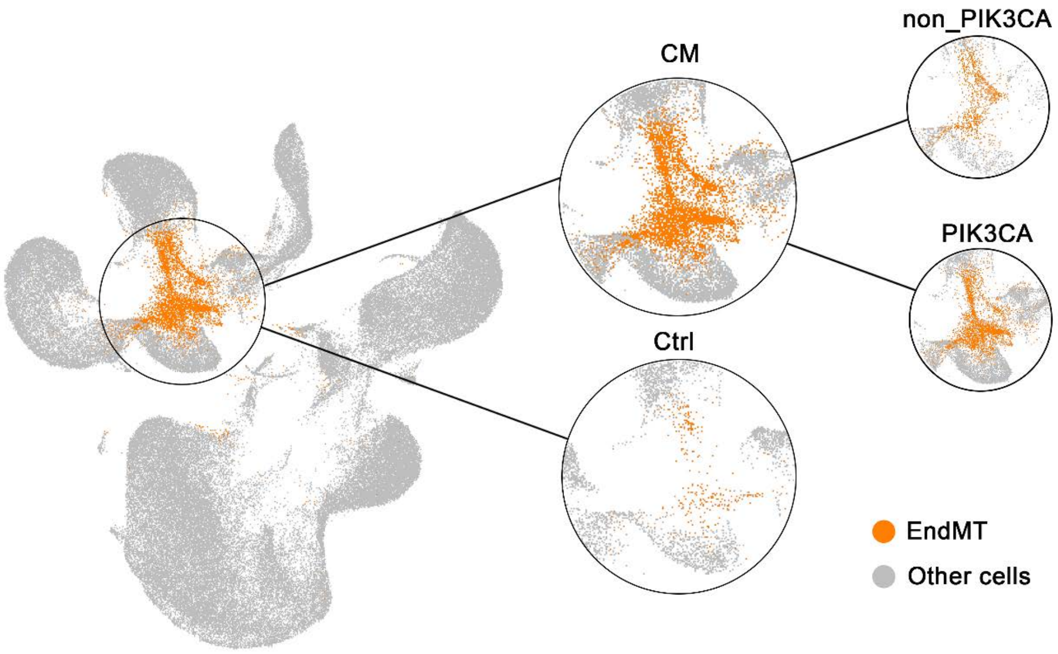

Supplementary Fig. 9. New marker genes and enrichment analysis of EndMT cells.

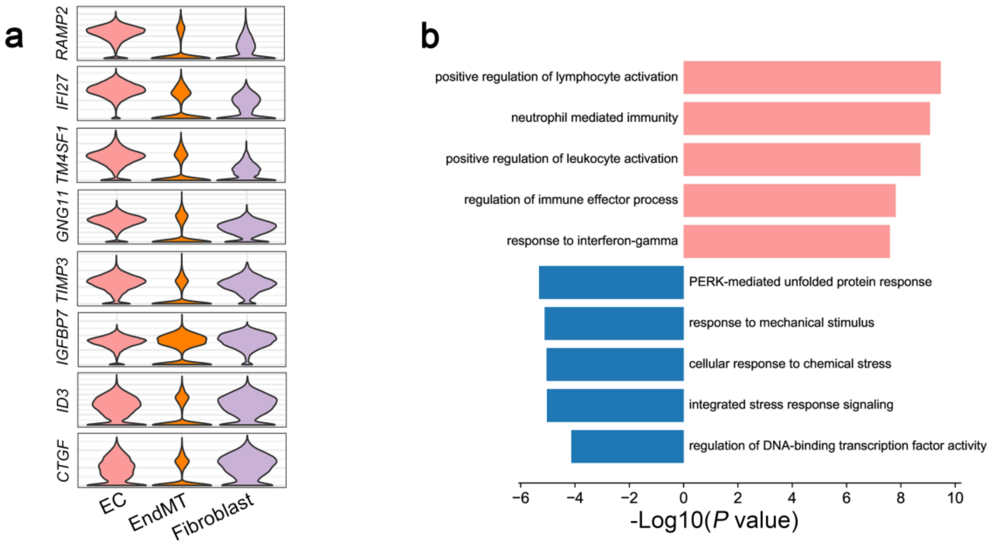

(a) New identified marker genes of EndMT cells shown in violin plots. (b) GO enrichment analysis terms of DEGs up-regulated (pink, avg_log2FC≥0.25 and p value<0.05) and down-regulated (blue, avg_log2FC≤-0.25 and p value<0.05) in lesion EndMT cells.

Supplementary Fig. 10. Expression of markers of ECs and SMCs in brain cells of mice after magnetic bead separation.

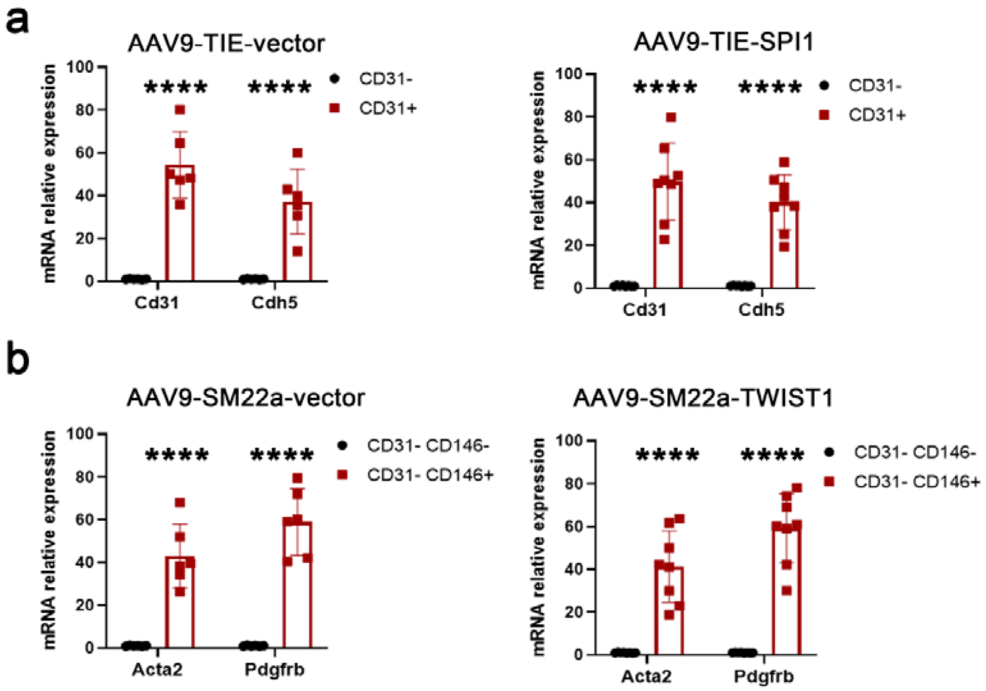

(a) Expression of EC markers (Cd31 and Cdh5) via RT-qPCR in CD31+ and CD31- cells using CD31 MicroBeads, mouse kit separation in AAV9-TIE-vector and AAV9-TIE-SPI1 group. (b) Expression of SMC markers (Acta2 and Pdgfrb) via RT-qPCR in CD31- CD146+ and CD31- CD146- cells using CD31 MicroBeads, mouse kit and CD146 MicroBeads, mouse kit separation in AAV9-SM22a-vector and AAV9-SM22a-TWIST1 group. Data were represented as mean±SD (AAV9-TIE-vector and AAV9-SM22a-vector group, n=6; AAV9-TIE-SPI1 and AAV9-SM22a-TWIST1 group, n=8). Statistics was performed using Student's t-test, and significance was determined as ****$P < 0.0001$. RT-qPCR primers were shown in Table S5.

Supplementary Fig. 11. UMAP plots showed four mural cell subclusters divided into control, CMs with *PIK3CA* gene mutations and CMs without *PIK3CA* gene mutations groups.

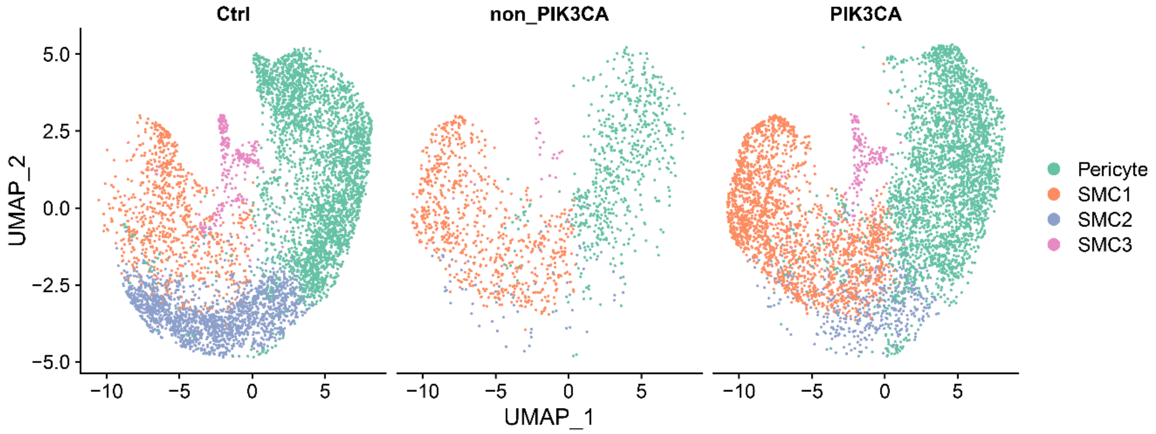

Supplementary Fig. 12. UMAP plots showed SMC1 and SMC2 subclusters divided into control and CMs groups.

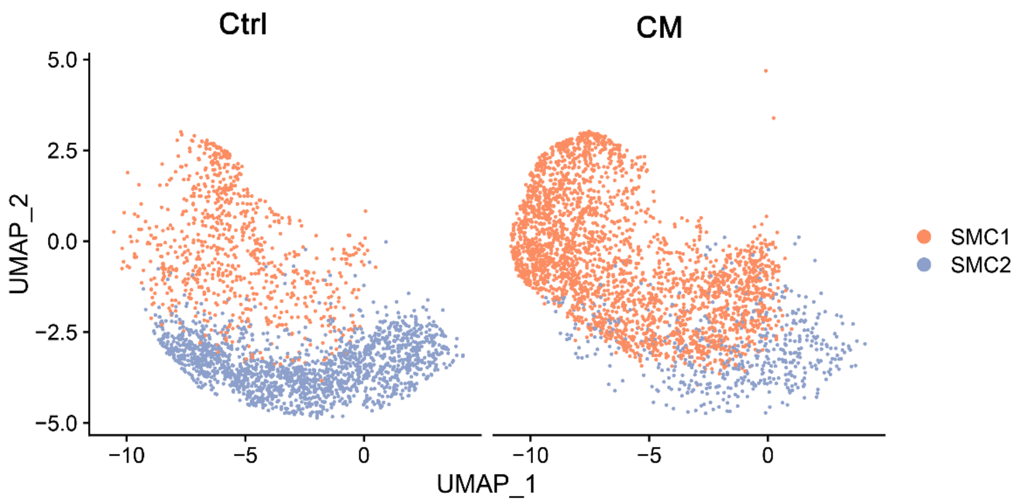

Supplementary Fig. 13. UMAP plots showed SMC1 and SMC2 subclusters divided into control, CMs with *PIK3CA* gene mutations and CMs without *PIK3CA* gene mutations groups.

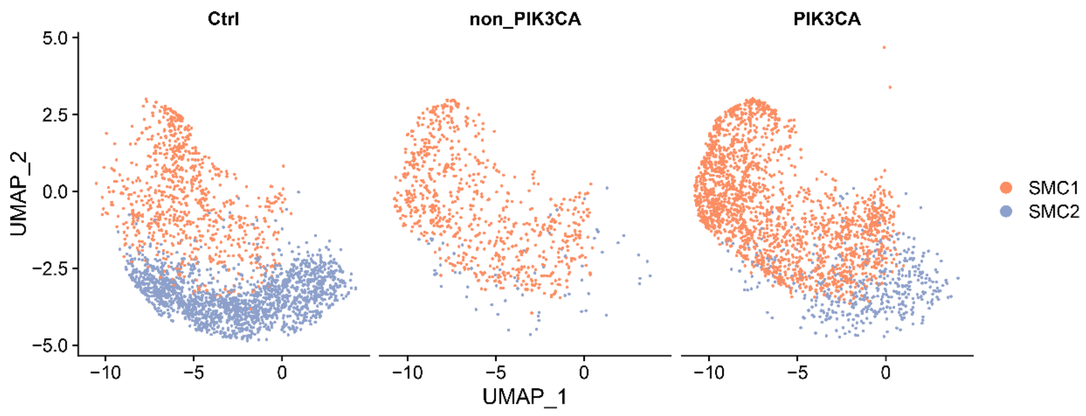

References

1. Hong, T. *et al.* Somatic MAP3K3 and PIK3CA mutations in sporadic cerebral and spinal cord cavernous malformations. *Brain* **144**, 2648-2658 (2021).
2. Orsenigo, F. *et al.* Mapping endothelial-cell diversity in cerebral cavernous malformations at single-cell resolution. *Elife* **9**, e61413 (2020).
